# GSTT2 Switches from a Homeostatic Antioxidant to a Cell-Cycle-Coupled Factor in Therapy-Resistant Esophageal Adenocarcinoma

**DOI:** 10.3390/cancers18193220

**Published:** 2026-10-06

**Authors:** Daysha Ferrer-Torres, Paramita Ray, Noah Etzioni, Max A. Hammer, Archismaan Ghosh, Meilan Liu, Vinay Jeeyar, Sachin Wani, Sunnie S. Kim, Benedetto Mungo, Stefan Marasligiller, Danielle Kim Turgeon, Jules Lin, Peter D. R. Higgins, Jason R. Spence, Kiran H. Lagisetty, David G. Beer, Dipankar Ray

**Affiliations:** 1 Department of Medicine, Division of Gastroenterology & Hepatology, University of Colorado Cancer Center, Anschutz Medical Campus, Aurora, CO 80045, USA; noah.etzioni@cuanschutz.edu (N.E.); sachin.wani@cuanschutz.edu (S.W.); 2 Katy O. and Paul M. Rady Esophageal and Gastric Center of Excellence, University of Colorado Cancer Center, Anschutz Medical Campus, Aurora, CO 80045, USA; sunnie.kim@cuanschutz.edu (S.S.K.); benedetto.mungo@cuanschutz.edu (B.M.); stefan.marasligiller@cuanschutz.edu (S.M.); 3 Department of Radiation Oncology, University of Michigan, Ann Arbor, MI 48109, USA; parray@umich.edu (P.R.); archisg@umich.edu (A.G.); lmiu@umich.edu (M.L.); vinayj@umich.edu (V.J.); 4 Department of Gastroenterology, University of Michigan, Ann Arbor, MI 48109, USA; max.hammer@ucsf.edu (M.A.H.); kturgeon@med.umich.edu (D.K.T.); spencejr@med.umich.edu (J.R.S.); 5 Department of Oncology, University of Colorado Cancer Center, Anschutz Medical Campus, Aurora, CO 80045, USA; 6 Department of Surgery, University of Colorado Cancer Center, Anschutz Medical Campus, Aurora, CO 80045, USA; 7 Department of Surgery, University of Michigan, Ann Arbor, MI 48109, USA; juleslin@med.umich.edu (J.L.); phiggins@med.umich.edu (P.D.R.H.); klagiset@med.umich.edu (K.H.L.); dgbeer2@gmail.com (D.G.B.)

**Keywords:** esophagus, cell proliferation, DNA damage response, GSTT2, FBXW7, APC-CDC20

## Abstract

**Background/Objectives:** Glutathione S-transferase theta 2 (GSTT2) is highly expressed in esophageal tissues from African American (AA) compared with European American (EA) individuals, but its localization, regulation, and relevance to esophageal adenocarcinoma progression remain unclear. **Methods:** Here, we combined FISH, immunofluorescence, patient-derived culture models, cell-cycle synchronization, biochemical perturbation, and transcriptomics to define GSTT2 expression in normal and neoplastic esophageal contexts. **Results:** In developing and adult esophageal tissues, GSTT2 mRNA and protein were broadly distributed across squamous epithelium and lamina propria, co-localizing with p63-positive basal/progenitor cells and extending into differentiated compartments, with no major ancestry-associated differences in spatial patterning. In Barrett’s esophagus, GSTT2 protein was retained in metaplastic columnar epithelium and slightly elevated in dysplastic versus adjacent non-dysplastic regions. GSTT2 mRNA was highest in normal squamous esophagus, reduced during Barrett’s-to-adenocarcinoma progression, but increased in treatment-resistant esophageal adenocarcinoma (EAC). In resistant EAC, GSTT2 co-expression shifted from homeostatic antioxidant programs to proliferation and mitotic pathways, including the G2-M checkpoint, E2F targets, and Reactome Cell Cycle, with leading-edge regulators CDK1 and CDC20. Sequence analysis identified putative APC-C and FBXW7 recognition motifs. In synchronized cells, GSTT2 abundance declined during S-G2/M and increased during mitotic exit/early G1 in cytosolic and membrane fractions. Forskolin, a dual inhibitor of FBXW7 and CDC20, stabilized GSTT2 and rescued CDC20- or FBXW7-associated loss, implicating APC-CDC20 and FBXW7 pathways in GSTT2 turnover. In EAC cells, by contrast, CDC20 was found to be a cooperative partner stabilizing GSTT2. **Conclusions:** These findings identify GSTT2 as a spatially widespread, ancestry-associated, cell-cycle-regulated protein with potential oncogenic roles in EAC resistance.

## Simple Summary

Esophageal adenocarcinoma is an aggressive cancer that often develops after years of acid and bile reflux damage to the esophageal lining. Healthy tissue depends on protective proteins to withstand this injury. One such protein, GSTT2, varies in abundance among individuals and populations, but its location, regulation, and role in cancer progression have remained unclear. We investigated where GSTT2 is found in esophageal tissue, how its expression is regulated, and how its function changes as healthy tissue progresses toward cancer and develops resistance to treatment. We found that GSTT2 normally helps protect the healthy esophagus but is lost during early tissue transformation. Surprisingly, it reappears in the most treatment-resistant tumors, where it takes on an altered, cancer-promoting role associated with cell division. These findings reveal GSTT2 as a context-dependent protein that shifts from protecting healthy tissue to supporting tumor growth and treatment resistance.

## 1. Introduction

Esophageal diseases, including Barrett’s esophagus and esophageal adenocarcinoma (EAC), represent major clinical challenges due to their increasing incidence, frequent late-stage diagnosis, and poor therapeutic response in advanced disease [1,2]. EAC typically develops through a metaplasia-to-dysplasia-to-adenocarcinoma sequence, often arising in the setting of chronic gastroesophageal reflux and Barrett’s esophagus [3]. Although genomic alterations such as *TP53* mutation, chromosomal instability, and dysregulated cell-cycle control are well-established features of EAC progression [4,5,6], the molecular factors that shape early esophageal epithelial biology and influence cancer susceptibility or therapy resistance remain incompletely understood. In addition, ancestry-associated molecular differences in the normal esophagus may contribute to variation in epithelial homeostasis, stress response, and disease biology, yet these differences remain understudied.

Chronic gastroesophageal reflux exposes esophageal tissue to acid and bile salts, which together generate high levels of reactive oxygen species (ROS) and oxidative DNA damage in epithelial cells [7,8,9]. This oxidative stress is a major driver of inflammation, impaired wound repair, and the progression from GERD to Barrett’s esophagus and ultimately esophageal adenocarcinoma. Our prior work identified GSTT2 (glutathione-S-transferase theta-2) as a protective detoxification enzyme abundant in the normal esophagus of African Americans (AA) who develop significantly less Barrett’s esophagus and esophageal adenocarcinoma when compared to European Americans (EA) [10]. GSTT2 conjugates glutathione to electrophilic and oxidative byproducts, reducing ROS burden and preventing downstream DNA damage [11]. Thus, in the normal esophagus, GSTT2 may function as a protective factor by limiting oxidative DNA damage. Prior transcriptomic analyses from our group demonstrated that GSTT2 mRNA and protein are expressed at higher levels in normal esophageal tissues from AA compared with EA individuals, suggesting that GSTT2 may represent an ancestry-associated component of esophageal epithelial biology. However, those studies were performed using whole-tissue biopsies, leaving unresolved whether GSTT2 expression is restricted to specific epithelial compartments, associated with basal progenitor populations, or present in non-epithelial stromal compartments.

Understanding the spatial localization of GSTT2 within the human esophagus is important because the squamous epithelium is organized into functionally distinct cellular compartments. TP63-positive basal cells support epithelial renewal and repair, while suprabasal and luminal cells undergo differentiation and barrier maturation [12,13]. Molecular differences within these compartments may influence epithelial injury responses, regenerative capacity, and susceptibility to transformation. Furthermore, the esophageal lamina propria contains stromal and immune-associated cell populations that can contribute to epithelial homeostasis and disease progression [13,14,15]. Thus, resolving GSTT2 expression at the cellular and tissue levels and the factors that maintain GSTT2 protein stability is necessary to define its potential biological function in both the normal esophagus and pre-malignant and malignant lesions.

Beyond its canonical role in detoxification, emerging evidence suggests that redox-regulatory proteins may intersect with core cell-cycle and cancer-associated pathways [16,17]. EAC is characterized by frequent *TP53* mutation and dysregulated cell-cycle progression, which can influence oxidative stress tolerance and therapeutic resistance [4,18,19]. The gain-of-function (GOF) mutant p53 has been reported to activate oxidative transcriptional programs in cancer contexts [20,21]. These interactions may allow tumor cells to tolerate elevated oxidative stress and survive cytotoxic therapy. Therefore, GSTT2 may function differently in normal esophageal epithelium compared with EAC, potentially shifting from a protective homeostatic enzyme to a contributor to tumor cell adaptation.

Protein abundance is also controlled post-translationally by ubiquitin-mediated degradation. Cell-cycle-regulated E3 ubiquitin ligases, including the anaphase-promoting complex/cyclosome (APC-C) and FBXW7-containing SCF complexes, coordinate the timely degradation of proteins required for orderly cell-cycle transitions [22,23]. APC-C, through its co-activators CDC20 and Fizzy-related 1/CDH1, regulates mitotic progression and exit, whereas FBXW7 targets multiple phosphorylated substrates involved in growth and proliferation and is further involved in DNA damage repair [24]. Dysregulation of these pathways is common in cancer and can alter the stability of proteins that influence cell survival, genome integrity, and therapy response. We noted the presence of putative APC-C and FBXW7 recognition motifs in GSTT2, raising the possibility that GSTT2 protein levels may be cell-cycle-regulated and that this regulation may be altered during malignant progression.

In this study, we integrate FISH, immunofluorescence, patient-derived culture, cell-cycle synchronization, mutant p53 expression, ubiquitin pathway perturbation, and transcriptomic analysis across the BE-dysplasia-EAC and treatment-resistant EAC spectrum to define the spatial, cellular, and regulatory features of GSTT2 in the human esophagus. We first mapped *GSTT2* mRNA and protein localization in developing, adult and Barrett’s esophagus human esophageal tissues. Using cell-cycle synchronization, mutant p53 expression systems, and ubiquitin pathway perturbation, we investigated mechanisms regulating GSTT2 abundance. Finally, we analyzed GSTT2 expression across Barrett’s esophagus, dysplasia, EAC, and treatment-resistant EAC datasets to determine whether GSTT2 expression is associated with cancer progression, DNA damage response, cell-cycle programs, and therapeutic resistance. Together, these studies provide a framework for understanding GSTT2 as a spatially widespread, ancestry-associated, and cell-cycle-regulated protein with context-dependent roles in normal esophageal biology and EAC pathogenesis.

## 2. Materials and Methods

**Cell lines and reagents.** Human embryonic kidney (HEK-293), cervical carcinoma (HeLa) and EAC cell lines (Flo1, Eso51) were purchased from the American Type Culture Collection (ATCC) and SigmaMillipore (Burlington, MA, USA). The cells were either grown in RPMI1640 or DMEM supplemented with 10% fetal bovine serum and antibiotics, as we previously reported [25]. The cells were routinely tested for any unwanted pathogen infection and genotyped for authenticity at the University of Michigan Advanced Genomics Core (AGC).

Antibodies included GSTT2 (sc-514667), Hsc70 (sc-7298) and EGFR (sc-03), which were from Santa Cruz Biotechnology (Santa Cruz, CA, USA). Cyclin B1 (D5C10, Cat. 12231), GAPDH (14C10, Cat. 2118), a-Tubulin (Cat. 2144), and HA (C29F4, Cat. 3724) (antibodies were purchased from Cell Signaling (Danvers, MA, USA) and anti-DDK (OTI4C5; Origene Technologies, Rockville, MD, USA). FuGENE HD (Promega Cat# E2311) and Lipofectamine 2000 (Cat# 11668019) were from ThermoFisher Scientific (Waltham, MA, USA). We used *CDH1 (Cat. M-005499-01-0005) SMARTpool* from Dharmacon (Lafayette, CO, USA) and the *FBXW7* pre-designed siRNA set (Cat. HY-RS04818) from MedChemExpress (Monmouth, NJ, USA). Human-specific *CDC20* siRNA (sc-42008) was purchased from Santa Cruz Biotechnology.

p53 (dominant negative R175H mutant)-pcw107-V5 was a gift from David Sabatini and Kris Wood (Addgene plasmid # 64638; http://n2t.net/addgene:64638 (accessed on 14 August 2026); RRID: Addgene 64638) [26], and pLenti6/V5-p53_R273H was a gift from Bernard Futscher (Addgene 22934; http://n2t.net/addgene:22934 (accessed on 14 August 2026); RRID: Addgene_22934) [27].

**Protein isolation, analysis and immunoblotting.** Proteins were isolated according to the protocol described in [28]. Briefly, a lysis buffer was prepared by mixing 50 mM HEPES/KOH, 150 mM NaCl, 1 mM EDTA, 2.5 mM EGTA, 1 mM N-ethylmaleimide, 1 mM NaF, 100 μM sodium orthovanadate, 10% glycerol, 10 mM β-glycerophosphate, 0.1% NP-40, and protease inhibitor cocktail. To prevent degradation of proteins, the entire procedure of extraction was carried out on ice, and the protein content was estimated using Bradford reagent. Gel electrophoresis was performed in 4–12% precast bis-tris gel (Invitrogen, Carlsbad, CA, USA), blotted on a PVDF membrane, and subjected to immunoblotting.

**Synchronization and cell fractionation.** HeLa cell synchronization was performed using double thymidine block and release, as we recently reported. Briefly, cells were maintained in 2 mM thymidine for 18 h, followed by a release in fresh media for 9 h. The cell cycle was then blocked again using the same concentration of thymidine for the next 15 h. Finally, the G1-synchronized cells were released again and collected every two hours. Cell lysates were also prepared and subjected to immunoblotting at each time point up to 14 h.

For cell fractionation, we used a subcellular protein fractionation kit (Cat. 78840, Thermo Scientific, Waltham, MA, USA), according to the manufacturer’s protocol.

**Sample collection.** Histologically normal, Barrett’s esophagus (BE), and tumor esophageal tissues were collected from consenting men and women who underwent upper endoscopy or surgical resection between 2017 and 2020 at the time of a scheduled inflammatory bowel disease screening, BE surveillance, or tumor surgical resection at the University of Michigan Health System (Michigan Medicine). Samples for this study were collected in accordance with the Declaration of Helsinki and approved by the Institutional Review Board (or Ethics Committee) of the University of Michigan under approved protocols: HUM00041845, HUM00103771, and HUM00176985.

Patient race was self-identified; following Ferrer-Torres et al. [29], white non-Hispanic individuals are referred to as European American (EA) and Black individuals as African American (AA). Fresh samples were collected in cold HYENAC medium and either processed immediately or cryopreserved and stored at −80 °C until use.

**Tissue preparation, immunofluorescence, and imaging.** Patient biopsies and tissues were fixed in 4% paraformaldehyde (Sigma-Aldrich, St Louis, MO, USA) overnight, washed with PBS, and dehydrated through a graded alcohol series: 30 min each in 25%, 50%, and 75% methanol in PBS/0.05% Tween-20, followed by 100% methanol, 100% ethanol, and 70% ethanol. The tissues were processed into paraffin using an automated tissue processor (Leica ASP300). Paraffin blocks were sectioned at 7 µm, and immunohistochemical staining was performed as previously described (Spence et al., 2011) [30]; Ferrer-Torres et al.) [29]. Briefly, slides were rehydrated through Histo-Clear, 100% ethanol, 95% ethanol, 70% ethanol, 30% ethanol, and deionized water (two changes of 3 min each). Antigen retrieval was performed in 1× sodium citrate buffer in a steamer for 20 min. The slides were washed in PBS, permeabilized for 10 min in 0.1% Triton X-100 in PBS, and blocked for 45 min in 0.1% Tween-20/5% normal donkey serum in PBS. Primary antibodies were diluted in blocking solution and applied overnight at 4 °C. The slides were washed three times in PBS, and secondary antibodies plus DAPI were applied for 60 min at room temperature, followed by three PBS washes and mounting in ProLong Gold (Thermo Fisher Scientific, Waltham, MA, USA).

**Fluorescence in situ hybridization** (**FISH**). FISH for *GSTT2* mRNA was performed on paraffin-embedded developing and adult esophageal tissue sections according to the manufacturer’s instructions. Sections were counter-stained with DAPI and imaged.

**Expression datasets.** *GSTT2* mRNA expression was analyzed in three microarray datasets. The GSE37203 cohort (Affymetrix HG-U133A, GPL96) provided 68 esophageal specimens spanning non-dysplastic BE (*n* = 9), BE with low-grade dysplasia (*n* = 7), LGD (*n* = 8), HGD (*n* = 7), chemotherapy-naïve EAC (*n* = 15), and therapy-resistant EAC (*n* = 22); diagnosis annotations were taken from the sample metadata. Normal esophageal tissue for the ancestry comparison was profiled in an independent cohort (AA/EA cohort, Affymetrix Gene ST) comprising 24 normal squamous samples (12 AA, 12 EA) from Ferrer-Torres et al. 2019. Malignant transformation was assessed in a third, independent esophageal/gastroesophageal cohort (Affymetrix Gene ST; Ferrer-Torres, Oncotarget 2016) [31] comprising normal squamous esophagus (*n* = 8), normal gastric mucosa (*n* = 5), and gastroesophageal adenocarcinoma (*n* = 35). Probe-level intensities were collapsed to gene symbols by averaging.

**Cross-platform normalization and group comparisons.** Because the normal tissue reference and the progression series were profiled on different microarray platforms, absolute log_2_ intensities are not directly comparable. To enable a common axis, *GSTT2* expression in each sample was expressed as its percentile rank among all genes measured in that sample (within-sample percentile rank), a platform-independent statistic (Supplemental Figure S1A). Differences across the six GSE37203 disease groups were assessed by the Kruskal–Wallis test; pairwise comparisons (therapy-resistant versus chemotherapy-naïve EAC; AA versus EA normal esophagus; and adenocarcinoma versus normal squamous esophagus in the transformation cohort) used the two-sided Mann–Whitney U test. Within the single-platform transformation cohort (Supplemental Figure S1B), *GSTT2* is reported as log_2_ intensity.

**GSTT2 co-expression and pathway over-representation.** Within each cohort, genes co-expressed with *GSTT2* were defined by Pearson correlation (r ≥ 0.5) and restricted to protein-coding genes (biotype annotation via MyGene.info) because the pathway databases used contain no annotations for non-coding or pseudogene loci. Over-representation analysis was performed with Enrichr against seven libraries (GO Biological Process, GO Molecular Function, GO Cellular Component 2023; KEGG 2021 Human; Reactome 2022; WikiPathways 2023 Human; MSigDB Hallmark 2020). Terms with Benjamini–Hochberg FDR < 0.05 were considered significant.

**Transcription factor co-expression screen.** Datasets: Two independent microarray datasets were analyzed. Normal esophagus included the Beer ancestry cohort (Affymetrix Human Gene ST array) comprising 40 normal esophageal biopsies (12 AA normal squamous, 12 EA) normal squamous, 8 AA Barrett’s-adjacent normal, and 8 EA Barrett’s-adjacent normal). Therapy-resistant esophageal adenocarcinoma (EAC) included the 22 therapy-resistant tumors from GSE37203 (Affymetrix HG-U133A, GPL96), a Barrett’s-to-EAC progression series in which the resistant group was annotated separately from 15 chemo-naïve adenocarcinoma, 9 Barrett’s, 8 low-grade dysplasia, 7 Barrett’s + LGD, and 7 high-grade dysplasia samples.

Probe-to-gene collapse: For the HG-U133A progression matrix, multiple probes mapping to the same gene symbol were collapsed to a single per-gene value by averaging (mean of probe-level log_2_ intensities), yielding a gene-level matrix of 13,212 genes × 68 samples. The 22 therapy-resistant tumors were selected from this matrix for the resistant-set analysis. The Beer Gene ST matrix was used at the gene level as provided.

Transcription factor annotation: The reference set of human transcription factors (TFs) was obtained from the curated human TF census [32] (humantfs.ccbr.utoronto.ca, v1.01), retaining the 1639 genes flagged as TFs. Of these, 1574 were present in the Beer Gene ST matrix and 1006 in the collapsed HG-U133A matrix.

Correlation analysis: For each dataset, Pearson and Spearman correlation coefficients between *GSTT2* log_2_ expression and every present transcription factor were computed across the samples of that group (normal esophagus, *n* = 40; therapy-resistant EAC, *n* = 22). Transcription factors with zero variance were excluded. *p*-values were adjusted for multiple testing by the Benjamini–Hochberg false discovery rate (FDR) procedure across all tested transcription factors within each dataset. Analyses were performed in Python version 3.11 (pandas, NumPy, SciPy, statsmodels).

**Patient-derived 2D cultures.** Patient-derived stem cell 2D cultures were established following Ferrer-Torres et al. 2022 [29]. In short, fresh samples were collected on cold HYENAC media and processed for culture; otherwise, biopsies were cryopreserved and stored at −80 °C until use, at which point they could be thawed and processed for generating new culture lines. To cryopreserve biopsies, tissue was minced into fine pieces and frozen using 1 mL/vial of HYENAC + 20% CBS serum and 10% DMSO. Cryovials were placed in Mr. Frosty’s at −80 overnight, then moved to liquid nitrogen tanks for long-term preservation. For culturing cell lines, we found that fresh biopsies could be processed immediately and grown at a 100% success rate. We observed that biopsy samples in HYENAC media could be kept for 24 h at 4 °C and grown successfully. Furthermore, samples could be shipped, at which point viable cell lines could still be robustly established.

**Immunofluorescence analysis of γ-H2AX.** PBS was removed from the fixed samples, and the protein–DNA was cross-linked with 500 µL of 10% phosphate-buffered formalin for 20 min at room temperature (RT). Cells were washed once with 500 µL TBS, followed by permeabilization with 100% cold methanol (−20 °C), then incubated for 1 h in a blocking buffer containing 5% goat serum, 1% BSA, and 0.2% Triton x100 in TBS (500 µL of blocking buffer per well). Each primary antibody (γ-H2AX 1:1500 (#05-636, Millipore) and GSTT2-1:500 (#514667, Santa Cruz)) was diluted in TBS with 1% BSA such that 150 µL was used for each cover glass and allowed to incubate on the cells overnight at 4 °C in a humidified chamber. The following day, the cells were washed 3 times with TBS-T for 5 min. The cells were then incubated with secondary antibody for 1 h at room temperature (1:50 Alexa Fluor 488 goat anti-mouse IgG1, #A21121, Life Technologies; 1:200 Alexa Fluor 594 goat anti-mouse IgG2a, #A21135, Life Technologies) (Carlsbad, CA, USA) in 1% BSA, followed by three 5 min washes with TBS-T. Coverslips were mounted onto slides using DAPI mounting solution (# P36935, Thermo Fisher, Waltham, MA, USA) and stored at −20 °C prior to microscopic and photographic imaging.

**Ki67/TP63 proliferation index.** Cultures were co-stained by multiplexed immunofluorescence for Ki67 and TP63, and TP63^+^/Ki67^+^ double-positive cells were quantified and normalized to total human nuclear antigen (HuNu)-positive nuclei (*n* = 4 patients: 2 AA, 2 EA; triplicate quantification per patient).

**Acid injury model.** Stratified 2D cultures were exposed once to pH 4 medium for 10 min to model the GERD-associated acid microenvironment using HCL to acidify the media. Twenty-four hours after injury, cells were split and replated at equal density (5 × 10^5^ cells/well), and the cell number was assessed 5 days post-injury using a cell counting Hemocytometer with trypan blue exclusion. DNA damage was quantified by γ-H2AX immunofluorescence 24 h post-injury in cultures co-stained for the basal marker KRT5, scoring the percentage of nuclei with >10 foci.

**Alkaline comet assay.** To determine H_2_O_2_-induced DNA damage, we used the alkaline comet assay as we recently described [25]. Briefly, frosted slides were coated with a layer of agarose and dried. About 2000 cells from different treatment groups were suspended in PBS and mixed with triple the volume of 1% low-melting-point (LMP) agarose at 37 °C and spread over the agarose-coated slides to make a thin layer. These slides were then kept in a cold room and allowed to solidify for four hours. The slides were then kept submerged in a highly alkaline lysis buffer (1.2 M NaCl, 100 mM Na_2_EDTA, 0.1% sodium lauryl sarcosinate, 0.26 M NaOH (pH > 13) overnight at 4 °C. The next day, the slides were rinsed three times at room temperature in alkaline rinse and electrophoresis solution (0.03 M NaOH, 2 mM Na_2_EDTA, pH 12.3) to remove excess salt from the lysis buffer. The slides were then placed onto a horizontal electrophoresis chamber and electrophoresed for 25 min at a voltage of 0.6 V/cm. The slides were then rinsed again with distilled water to remove salt. The gels on the slides were stained with 2.5 μg/mL of propidium iodide (PI) for 20 min, rinsed once with distilled water, dried overnight, and imaged. For each treatment, 50 comet images were captured, and the olive tail moments were calculated using Comet Assay IV^TM^, Instem software (Staffordshire, UK). The average tail moment was calculated for the control and different treatment groups at 24 h post-treatment and subjected to statistical analysis.

**Quantification and statistical analysis.** GraphPad Prism (11.1.0 for Windows and Mac, released on 19 August 2026) was used to conduct statistical analyses and generate plots. Tests are specified per experiment above and in the figure legends. A significance threshold of *p* < 0.05 was applied throughout; exact *p*-values are reported in the figures. Unless otherwise stated, cell-based experiments were performed in two to three independent experiments with two to three technical replicates, and data are presented as mean ± s.d.

## 3. Results

**Figure 1:**
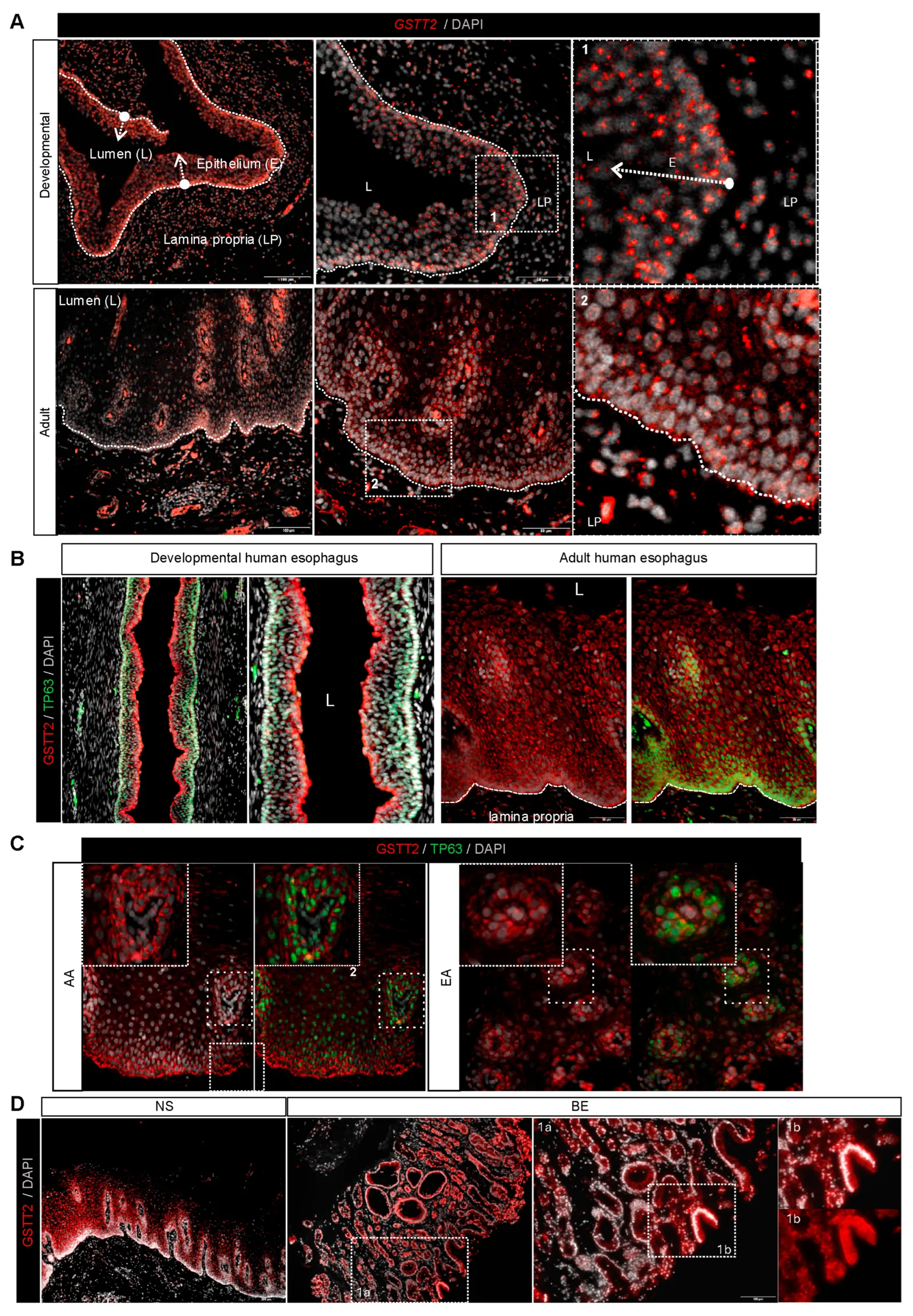
**Spatial localization of *GSTT2* mRNA and protein in developing and adult human esophageal tissues and spatial expression patterns in African American versus European American tissues and Barrett’s esophagus.** (**A**) Fluorescent in situ hybridization of esophageal tissue sections of early developmental vs. adult esophageal tissues. (**B**) Co-immunofluorescence staining for GSTT2 (red), the basal/progenitor cell marker TP63 (green), and DAPI (grey) in developing (**left**) and adult (**right**) human esophageal tissues. GSTT2 and TP63 signals co-localize within the epithelial layer in both the developmental and adult contexts, demonstrating expression of GSTT2 in TP63-positive basal cells. In both, GSTT2 was expressed in the full-thickness stratified epithelium as well as the lamina propria. In early development, there are high levels of GSTT2 expression in the ciliated-luminal compartment. (**C**) Co-immunofluorescence staining for GSTT2 (red), TP63 (green), and DAPI (grey) in esophageal biopsy tissues from African American (AA, (**left**)) and European American (EA, (**right**)) subjects. Dashed boxes indicate regions shown at higher magnification in adjacent panels. (**D**) GSTT2 protein (red) with DAPI (grey) in normal squamous esophagus (NS) and Barrett’s esophagus (BE). In insets 1a–1b, higher-magnification views of BE dysplastic regions display increased GSTT2 signal relative to non-dysplastic metaplastic regions. AA, African American; EA, European American; E, epithelium; LP, lamina propria; L, lumen.

***GSTT2 protein localizes throughout the esophageal epithelium***. We previously reported that GSTT2 is overexpressed in the esophagus of AAs when compared to EAs [10]. Nonetheless, this was performed on whole-tissue biopsies, and the spatial characterization of GSTT2 in the human esophagus remains unknown. Furthermore, characterization of GSTT2 expression in Barrett’s esophagus is unknown. Therefore, to characterize the spatial distribution of GSTT2 mRNA and protein within the human esophagus and in Barrett’s esophagus, we performed fluorescence in situ hybridization (FISH) and immunofluorescence staining on developing, adult, and Barrett’s esophagus human tissue sections, as well as in a cohort of AA vs. EA tissue sections (Figure 1). In the developing human (post-conception date 130) and adult esophageal tissues, *GSTT2* probes were detected in a broad, diffuse pattern throughout the full thickness of the squamous epithelium, extending from the basal layer to the lumen, with signal also present in the underlying lamina propria (Figure 1A, top and bottom panels). When looking at the protein localization of GSTT2 within the early vs. adult human esophageal tissues, we observed high expression in the ciliated luminal population, as well as in the stratified epithelium and the lamina propria (Figure 1B, left panels). GSTT2 expression in the adult human esophagus was observed throughout the full thickness of the stratified epithelium, as well as in the lamina propria (Figure 1B, right panel).

To determine whether GSTT2 expression is associated with the basal progenitor cell compartment, we co-stained tissue sections with antibodies against GSTT2 and the established basal/stem cell marker TP63. In both the developing and adult esophageal tissues, GSTT2 (red) and TP63 (green) signals co-localized within the epithelium, demonstrating that GSTT2 is expressed in TP63-positive basal cells (Figure 1B). This co-localization was observed across the full length of the epithelial mucosa in the developing esophagus and at the basal layer of the adult esophageal epithelium, adjacent to the lamina propria. Because we previously identified GSTT2 as differentially expressed in the esophagus of AA vs. EA, we compared the spatial distribution of GSTT2 in these two groups. We could not detect any differences in spatial patterns of expression between the two groups, suggesting GSTT2 localized expression is similar in both groups (Figure 1C).

Having established the spatial distribution of GSTT2 in the normal esophagus, we next asked whether GSTT2 expression is detected in BE tissues with matched normal squamous (NS) (Figure 1D). In NS, GSTT2 was expressed throughout the squamous epithelium and lamina propria, consistent with the pattern described above (Figure 1A–C). In BE, however, GSTT2 signal was confined to within metaplastic columnar tissue (Figure 1D). Interestingly, within individual BE specimens, GSTT2 expression was not uniform: regions harboring dysplasia showed higher GSTT2 expression than adjacent non-dysplastic metaplastic regions of the same tissue (Figure 1D, insets 1a–1b). Altogether, we found that GSTT2 is expressed from development to the adult esophagus. It is expressed in both epithelial and lamina propria cells, and within BE tissues, we observed dysplastic regions with higher expression.

***GSTT2 mRNA is abundant across progression and rises specifically in therapy-resistant EAC.*** To place these tissue in vivo observations on a quantitative axis of patient samples at the transcription level, we examined *GSTT2* mRNA across the full histological spectrum of esophageal neoplasia in the GSE37203 cohort (*n* = 68): non-dysplastic BE (*n* = 9), BE with low-grade dysplasia (*n* = 7), low-grade dysplasia (LGD, *n* = 8), high-grade dysplasia (HGD, *n* = 7), chemotherapy-naïve EAC (*n* = 15), and therapy-resistant EAC (*n* = 22). Because the normal esophagus reference and the progression series were profiled on different microarray platforms, we expressed *GSTT2* as its within-sample percentile rank across the transcriptome, a platform-independent measure (Supplemental Figure S1A). Relative to normal squamous esophagus (median 57th percentile), *GSTT2* rank fell across BE-to-EAC progression (Barrett’s through chemotherapy-naïve EAC; percentile medians 16–21) and rose specifically in therapy-resistant tumors (median 25th percentile; Kruskal–Wallis *p* = 1.1 × 10^−5^ across disease groups; resistant versus chemotherapy-naïve EAC, Mann–Whitney *p* = 1.2 × 10^−4^; Supplemental Figure S1A). Nonetheless, it is apparent that four HGD and two EAC-naïve tissues are above the mean, suggesting a potential earlier increased *GSTT2* expression in these tissue types (Supplemental Figure S1A). To corroborate the reduced expression of *GSTT2* in adenocarcinomas relative to normal squamous, we examined a dataset from an independent patient cohort comprising normal squamous esophagus, normal gastric mucosa, and gastroesophageal adenocarcinoma [31]. *GSTT2* was highest in normal squamous esophagus (median 6.4 log_2_) and significantly lower in adenocarcinoma (median 4.2 log_2_; Mann–Whitney *p* = 0.013), with normal gastric mucosa being intermediate (Supplemental Figure S1B). Therefore, *GSTT2* is not progressively induced along the metaplasia → dysplasia → adenocarcinoma sequence; rather, it is expressed at high levels in normal squamous epithelium, expressed at lower levels in adenocarcinoma development, and selectively increased in the therapy-resistant state.

**Figure 2:**
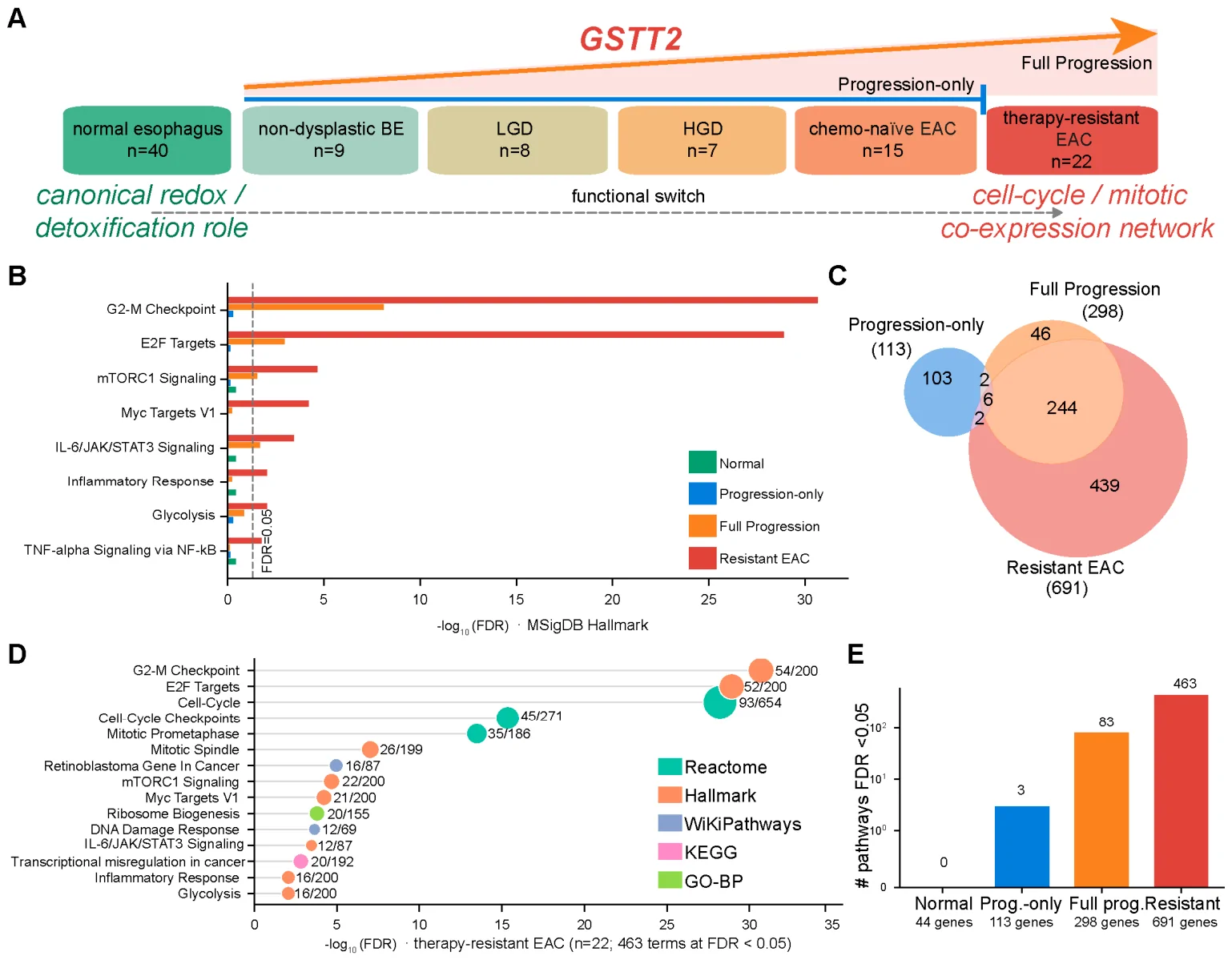
**GSTT2 co-expression switches to a proliferative program in therapy-resistant EAC.** Genes co-expressed with *GSTT2* (Pearson r ≥ 0.5) were identified within each cohort and tested for pathway over-representation. (**A**) Schematic of the disease axis. (**B**) Hallmark pathway enrichment (−log^10^ FDR) across cohorts; G2-M checkpoint and *E2F* targets rise specifically in resistant EAC. (**C**) Membership of *GSTT2*-correlated genes across progression and resistance EAC cohorts. (**D**) Top enriched pathways in resistant EAC. (**E**) Number of significantly enriched terms (FDR < 0.05) per cohort: 0 (normal), 3 (progression only), 463 (resistant EAC). FDR—false discovery rate; #—number.

***GSTT2* co-expression switches to a proliferative, cell-cycle program in therapy-resistant EAC.** We then asked what transcriptional program accompanies *GSTT2* in each disease state. Therefore, we identified genes co-expressed with *GSTT2* (Pearson r ≥ 0.5) within each cohort and tested them for pathway over-representation (Figure 2). In the normal esophagus, no pathway showed statistical significance after multiple testing correction (0 terms at FDR < 0.05); nonetheless, oxidative stress was observed as one of the trending pathways (Supplemental Table S1). In the BE-to-EAC progression cohort, with resistant tumors excluded, analyses yielded only three significant terms (UV Response Dn, TFAP2 family, basolateral plasma membrane) (Supplemental Table S2), none implicating cell-cycle machinery (Figure 2A,B, Supplemental Figure S2). In striking contrast, the *GSTT2* co-expression network in therapy-resistant EAC was dominated by proliferation and cell-cycle programs: Hallmark G2-M Checkpoint (54/200 genes, FDR = 2 × 10^−31^), *E2F* targets (52/200, FDR = 1 × 10^−29^), and Reactome Cell Cycle (93/654, FDR = 6 × 10^−29^) (Figure 2B,D, Supplemental Table S3). The leading-edge genes included the mitotic regulators *CDK1*, *CDC20*, *CCNB1*, *BUB1*, and *UBE2C* (Figure 2C), and the number of significantly enriched terms increased from 0 (normal) to three (progression only) to 463 (resistant EAC) (Figure 2E). This proliferative signature was contributed almost entirely by the 22 therapy-resistant tumors rather than by the Barrett’s-to-EAC progression dataset, identifying a resistance-specific functional switch, with *GSTT2* becoming coupled to the mitotic machinery.

A prior study using prostate cancer cells reported that *GSTT2* expression can be regulated by a redox-regulated transcription factor, NRF2 (encoded by *NFE2L2*) [33]. Analysis of the gene expression data, however, revealed no correlation between *NFE2L2* and *GSTT2* in the normal esophagus, during progression into non-dysplastic BE, LGD, HGD, treatment-naïve EACs and even in treatment-resistant EACs (Supplemental Figure S3). Such data led us to hypothesize that the induction of GSTT2 noted in treatment-resistant EACs may possibly be attributed to NRF2-independent transcription and post-translational stabilization of GSTT2 protein.

Based on gene expression data in normal esophageal tissues (*n* = 40), we found that 11 transcription factors were highly correlated with *GSTT2* (Supplemental Figure S4A). Among them, *ZBTB32* was most correlated (r = 0.58; *p* = 9.9 × 10^−5^) (Supplemental Figure S4B). As the AA population is known to express higher levels of GSTT2 relative to the EA population, *ZBTB32* expression also showed higher levels in the AA normal esophagus. In treatment-resistant EACs (*n* = 22), *GSTT2* transcript expression was most correlated with *FOXN2* (r = 0.82; *p* = 3.4 × 10^−6^) (Supplemental Figure S4C,D), suggesting a switch in gene expression during therapy resistance.

**Figure 3:**
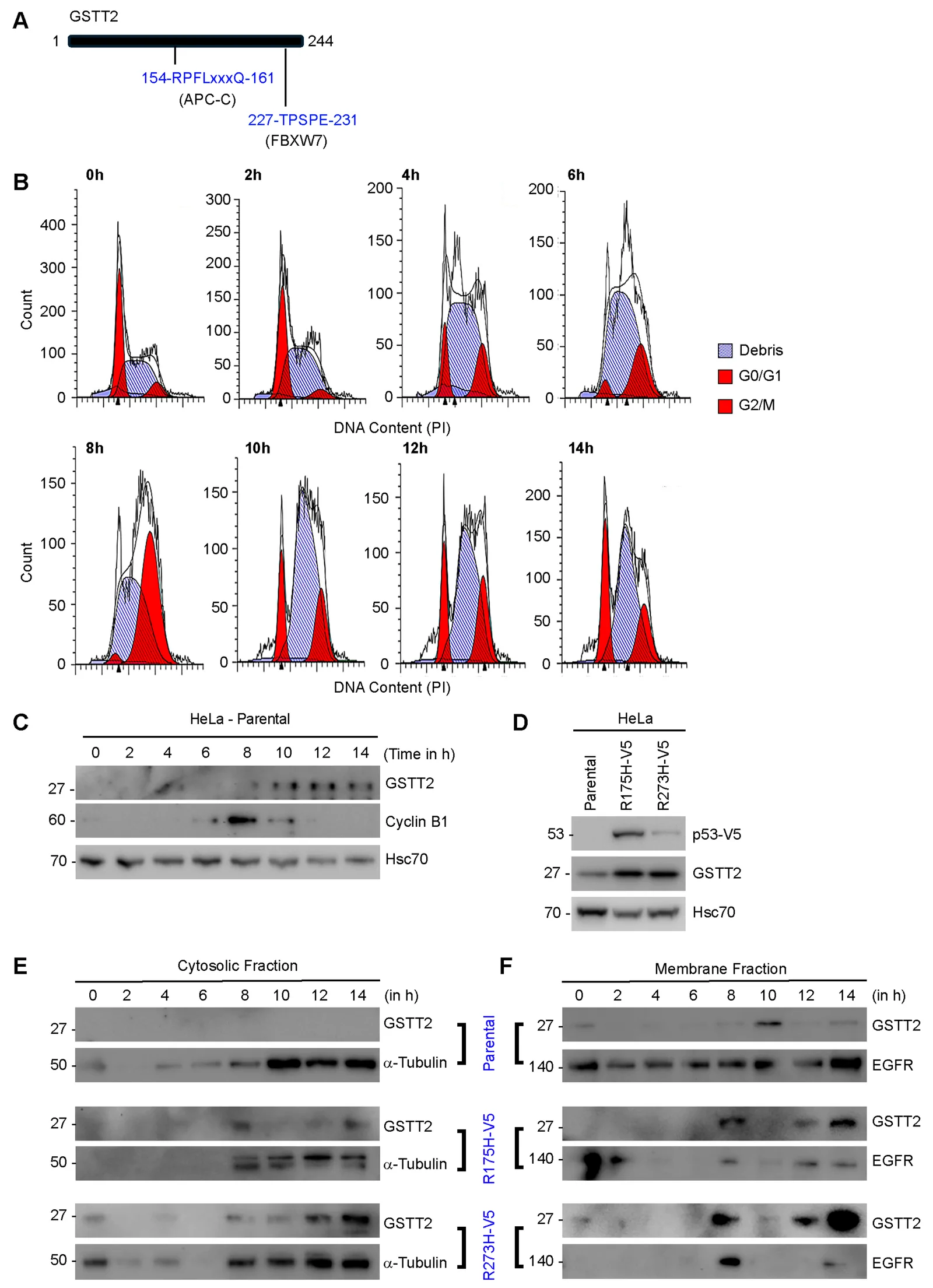
**GSTT2 is a cell-cycle-regulated protein.** (**A**) Putative APC-C and FBXW7 binding domains are present in GSTT2 protein. (**B**) HeLa cells were subjected to cell synchronization studies using double thymidine block and release. Following synchronization, cells were harvested every two hours, fixed, and subjected to FACS analysis. (**C**) Protein lysates were also prepared from another set of plates and subjected to immunoblotting using the indicated antibodies. (**D**) HeLa cells were infected with lentivirus expressing either p53-R175H-V5 or -R273H-V5 transgene, and transduced cells were selected using puromycin and G418, respectively, to obtain stable isogenic lines. Cell lysates from the three isogenic lines were subjected to immunoblotting using the indicated antibodies. (**E**,**F**) The above isogenic HeLa cells were first synchronized and collected at the indicated time points. Cells were then subjected to fractionation as detailed in the Section 2. Cytosolic and membrane fractions were then subjected to immunoblotting, as shown. Alpha-tubulin was used as a cytosolic marker, and EGFR as a membrane marker.

**GSTT2 contains putative APC-C and FBXW7 recognition motifs and is regulated during cell-cycle progression.** To understand the importance of post-translational regulation of GSTT2 protein, we analyzed and identified two putative degrons: 154-RPFLAGQQ-161 and 227-TPSPE-231. These motifs may be recognized by two distinct classes of E3 ubiquitin ligases: the anaphase-promoting complex/cyclosome (APC-C) and FBXW7, respectively (Figure 3A). Because both E3 ligases play critical roles in cell-cycle regulation, we hypothesized that GSTT2 may contribute to, or be regulated during, cell-cycle progression. To test this possibility, we used human cervical carcinoma HeLa cells, a wild-type p53-containing and GSTT2-expressing cell line that is widely used in cell-cycle studies (Figure 3B). Cells were synchronized using a double thymidine block-and-release protocol, as we previously standardized [34]. Following release, we observed increased GSTT2 expression in cells exiting mitosis and entering early G1, as determined by DNA-content analysis using FACS and by cyclin B1 expression (Figure 3C).

As HeLa cells contain wild-type p53, and the presence of mutant p53 is prevalent (>70%) in the majority of EACs, we generated isogenic HeLa cell lines expressing different mutant p53 transgenes, particularly the Arg175His (R175H) and Arg273His (R273H) missense mutants commonly seen in EAC patients. The ectopic expression of mutant p53 was confirmed using the V5 tag (Figure 3D). Notably, mutant p53 expression in HeLa cells further increased GSTT2 levels compared with wild-type p53-containing parental HeLa cells. GSTT2 is predominantly a cytosolic enzyme, although under certain conditions, it can be detected in the membrane fraction [35]. To further examine the effect of mutant p53 on GSTT2 cell-cycle distribution and subcellular localization, we performed cell synchronization followed by subcellular fractionation in parental HeLa cells and in the two mutant p53-overexpressing isogenic lines. As shown in Figure 3E,F, compared to parental HeLa cells, GSTT2 expression was readily detected in mutant p53-expressing isogenic HeLa cells, and the kinetics were comparable in the cytosolic and membrane fractions, with lower expression during S–G2 phases and higher expression as cells exited mitosis and entered G1. Together, these findings indicate that GSTT2 is a cell-cycle-regulated protein expressed during mitotic exit and early G1 phases.

**Figure 4:**
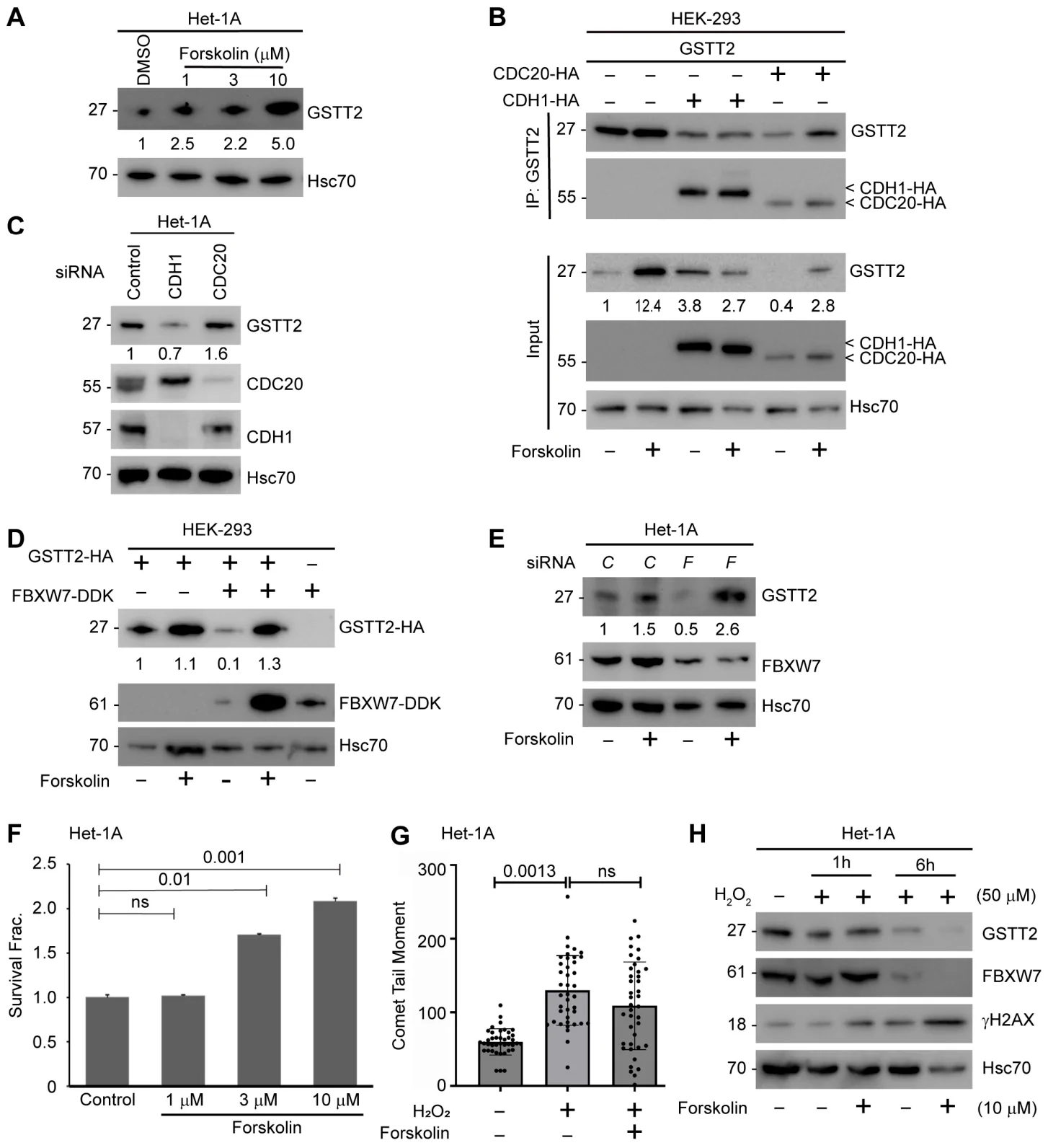
**GSTT2 protein level is influenced by APC-CDC20 and/or FBXW7.** (**A**) Het-1A cells were treated with different concentrations of forskolin as indicated. Twenty-four hours post-treatment, cells were harvested, and cell lysates were subjected to immunoblotting using the indicated antibodies. (**B**) Human embryonic kidney (HEK-293) cells were transfected with HA-tagged GSTT2 along with either CDH1 or CDC20 (as indicated). Twenty-four hours post-transfection, cells were treated with 10 μM forskolin for an additional 24 h. Cell lysates were then harvested, followed by immunoblotting using GSTT2 antibody and immunoblotted as shown. Total cell lysates were used as inputs. (**C**) Het-1A cells were transfected with either non-specific (control), CDH1 or CDC20 siRNAs as indicated. Forty-eight hours post-transfection, cell lysates were prepared and immunoblotted as shown. (**D**) HEK-293 cells were transfected with GSTT2-HA along with DDK-tagged FBXW7 as shown. Twenty-four hours post-transfection, cells were treated with forskolin as above and collected after 24 h post-treatment. Cell lysates were subjected to immunoblotting as indicated. (**E**) Het-1A cells were subjected to siRNA transfection using either control or FBXW7-specific siRNAs as indicated. Forty-eight hours post-transfection, cells were treated with forskolin as above, and 24 h post-treatment, cell lysates were prepared and immunoblotted. (**F**) Het-1A cells were plated in triplicate at a clonal density and, 24 h post-plating, treated with different doses of forskolin. Cells were left for the clonogenic survival assay, as detailed in the Section 2. Survival fraction (SF) was calculated for each treatment setting, considering the DMSO-treated condition as ‘1’. Similar experiments were performed three independent times, and statistical significance was calculated by unpaired t-tests (1 μM: ns; 3 μM: *p* = 0.019; 10 μM: *p* = 0.0012). Error bars represent mean ± SD. (**G**) Het-1A cells were treated with 50 μM H_2_O_2_, and cells’ ability to repair damaged DNA was determined at 24 h post-treatment using the alkaline comet assay. The results show quantification of olive tail moment (*n* = 50 cells) and repair defects upon H_2_O_2_ damage and subsequent treatment with forskolin (10 μM). The statistical significance of differences between control, H_2_O_2_, and H_2_O_2_ + forskolin-treated groups was evaluated using 2-way ANOVA, and *p*-values are included. (**H**) Cell lysates from the above study were also harvested and subjected to immunoblotting using the indicated antibodies.

To evaluate the functional relevance of the D-box and phosphodegron motifs in GSTT2, we treated cells with forskolin, a plant-derived labdane-type diterpenoid that activates adenylate cyclase and increases intracellular cyclic AMP (cAMP) levels [36]. Elevated cAMP activates both protein kinase A (PKA) and extracellular signal-regulated kinase (ERK) [37,38], which can phosphorylate APC-C subunits [39] and FBXW7 [40], respectively, thereby inhibiting their E3 ligase activities. Consistent with this model, forskolin increased GSTT2 levels in Het-1A cells in a dose-dependent manner, suggesting that APC-C-GSTT2 and FBXW7-GSTT2 regulatory pathways may contribute to GSTT2 turnover (Figure 4A).

APC-C is a multi-subunit E3 ubiquitin ligase that uses either Fizzy-related 1/CDH1 or CDC20 as substrate recognition adaptor proteins in a cell-cycle-stage-dependent manner [41]. APC-CDH1 primarily recognizes substrates during G1/S, whereas APC-CDC20 functions predominantly during mitosis. Both complexes recognize substrates through the consensus destruction box (D-box) motif, RxxLxxxN/D/E [41]. Because GSTT2 expression was enriched during mitotic exit and early G1 but reduced during S and G2/M phases (Figure 3), we hypothesized that GSTT2 may be regulated by APC-CDC20 during G2/M. To test whether GSTT2 interacts with APC-C, we performed immunoprecipitation followed by immunoblotting. GSTT2 was detected in immunocomplexes containing both APC-CDH1 and APC-CDC20 (Figure 4B). However, CDC20 and CDH1 had opposing effects on GSTT2 abundance: CDC20 overexpression decreased GSTT2 levels, whereas CDH1 overexpression increased GSTT2 expression. Furthermore, forskolin treatment rescued CDC20-mediated GSTT2 degradation. These observations are consistent with the GSTT2 expression kinetics observed during cell-cycle synchronization and suggest that APC-CDC20 functions as a novel E3 ligase promoting GSTT2 degradation during the G2/M phase of the cell cycle. Conversely, *CDC20* loss increased GSTT2 levels, whereas CDH1 loss decreased GSTT2 levels (Figure 4C).

As GSTT2 expression is low in S-phase cells, we further hypothesized that it may be regulated by FBXW7, an E3 ligase known to be active in S-phase cells. As expected, FBXW7 overexpression resulted in reduced GSTT2 expression, which was rescued by forskolin treatment (Figure 4D). Interestingly, forskolin also increased FBXW7 levels, possibly by inhibiting FBXW7 autoubiquitination and degradation. Unexpectedly, however, *FBXW7* loss also destabilized GSTT2 (Figure 4E). Because FBXW7 loss is known to indirectly influence CDC20 levels [42], we hypothesize that following the loss of FBXW7, CDC20 may be upregulated to cause GSTT2 loss. Thus, forskolin, which inhibits CDC20, rescued GSTT2 levels in the absence of FBXW7. FBXW7 is a well-established tumor suppressor that regulates several critical cell-cycle proteins, including cyclin E [43]. Loss of FBXW7 or E3 ligase-inactivating mutations have been reported in multiple cancer types [44]. Analysis of cBioPortal datasets across gastroesophageal cancers revealed deletions and mutations in FBXW7, suggesting that inactivation of this pathway may promote GSTT2 protein stabilization in EACs (Supplemental Figure S5A). For this E3 ubiquitin ligase, we did not notice a correlation in gene expression between GSTT2 and FBXW7 in normal esophageal cells or during EAC progression and therapy resistance (Supplemental Figure S5B). At the protein level, however, we performed dual immunofluorescence staining of GSTT2 and FBXW7 in both the human fetal esophagus and adult esophagus and noted the co-localized expression of both proteins in these tissues (Supplemental Figure S5C,D).

To determine the functional consequence of forskolin-mediated GSTT2 induction, we performed clonogenic assays in Het-1A cells. Forskolin treatment increased clonogenic survival (Figure 4F), suggesting that GSTT2 induction may enhance cell survival capacity. Because GSTT2 has been implicated in protection against DNA damage [24], we next treated cells with hydrogen peroxide (H_2_O_2_) and assessed whether forskolin could protect the cells from DNA damage using the alkaline comet assay. As shown in Figure 4G, forskolin produced a non-significant trend toward improved DNA repair. We hypothesize that this limited effect may reflect the G1-specific expression pattern of GSTT2, a phase in which non-homologous end joining (NHEJ) predominates, whereas H_2_O_2_-induced double-strand breaks are repaired primarily through homologous recombination repair (HRR). In addition, H_2_O_2_-mediated GSTT2 downregulation was not rescued by forskolin, further supporting the minimal effect of forskolin on DNA damage repair under these conditions (Figure 4H).

**Figure 5:**
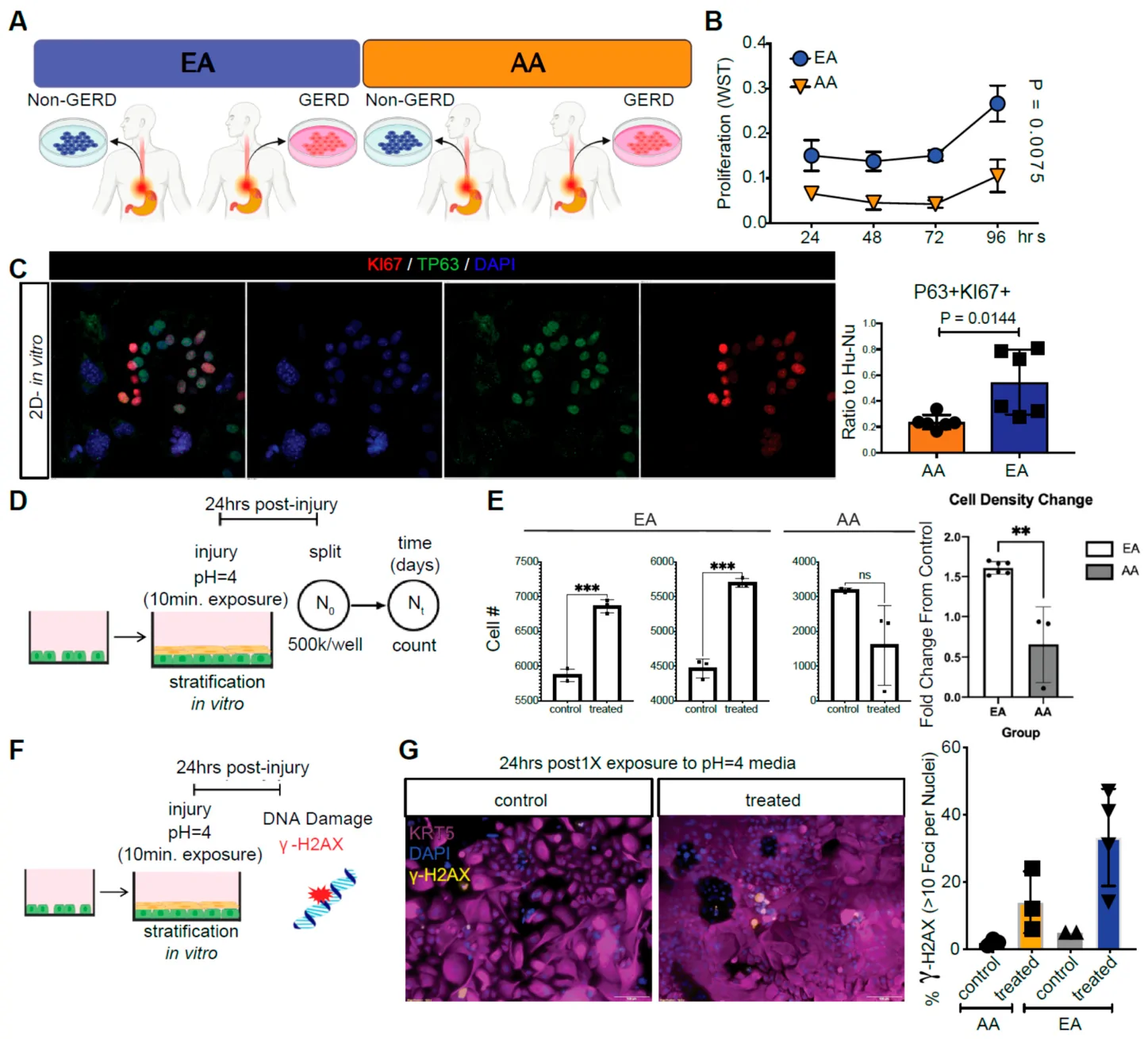
**Injury response is significantly different between AA and EA cells.** (**A**) We derived 2D esophagus cultures from AA and EA GERD and Non-GERD patients. (**B**) Cultures derived from GERD AA vs. EA show a significant difference in the endogenous proliferation rate of the cells, with EA having a higher proliferative rate when compared to AA (*p* = 0.00075). (**C**) Ki67 protein was used to validate the endogenous proliferative status between EA and AA cells, showing a significant difference in Ki67^+^ between AA and EA. *n* = 4 total patients (*n* = 2 AA vs. *n* = 2 EAs) (quantitation data is shown in triplicate per patient). (**D**,**E**) Upon stratification and injury exposure, EA cells show a significant differential response, with higher proliferation and accumulation of DNA damage when compared to AA cells. (**F**,**G**) EA esophageal KRT5^+^ (purple) cells exposed to injury with low-pH media (pH = 4) accumulate more DNA damage, as measured by γ-H2AX, when compared to AA cells. ** *p* ≤ 0.001; *** *p* ≤ 0.0001; ns, not significant (*p* > 0.05).

**Injury response is significantly different between AA and EA esophageal basal stem cells.** To investigate whether ancestral background influences the esophageal epithelial response to acid injury, we established 2D esophageal epithelial cultures derived from AA and EA patients with and without gastroesophageal reflux disease (GERD) (Figure 5A). Baseline proliferation assessed by WST assay over a 96 h time course revealed that GERD-associated EA cultures exhibited significantly higher endogenous proliferative capacity compared to AA-derived cultures (*p* = 0.0075; Figure 5B). This finding was validated at the protein level using multiplex immunofluorescence for the proliferation marker KI67 in combination with the basal epithelial marker TP63. Quantification of TP63^+^/KI67^+^ double-positive cells, normalized to total human nuclear antigen (HuNu), confirmed significantly greater proliferative activity in EA cultures relative to AA cultures (*p* = 0.0144; Figure 5C).

To model the esophageal acid microenvironment associated with GERD-mediated injury, stratified 2D cultures were subjected to a single 10 min exposure to pH 4 media; 24 h after injury, they were split and replated at equal density (500 k/well) and assessed for cell numbers 5 days post-injury (Figure 5D). Cell counts following acid exposure revealed that EA-derived cultures from two independent patient lines (HT227 and HT180) exhibited a significant increase in cell number relative to untreated controls (*p* < 0.001), while the AA-derived line (HT261) showed no significant change in cell density following injury (Figure 5E). Fold-change analysis confirmed a significantly greater proliferative response to acid injury in EA compared to AA cultures (*p* < 0.01; Figure 5E, right panel).

To assess whether differential proliferative responses were accompanied by differences in genomic integrity, we quantified DNA double-strand break accumulation using gamma-H2AX (γ-H2AX) immunofluorescence 24 h post-injury in stratified cultures co-stained with the basal marker KRT5 (Figure 5F,G). EA-derived cultures showed a marked increase in the percentage of nuclei with greater than 10 g-H2AX foci following acid exposure compared to untreated controls, while AA cultures demonstrated a comparatively modest injury-associated DNA damage response (Figure 5G). Together, these data indicate that EA and AA esophageal epithelial cells are distinct in their proliferative capacities and in their genomic responses to acid-mediated injury, with EA cells accumulating a more significant but potentially genotoxic regenerative response.

**Figure 6:**
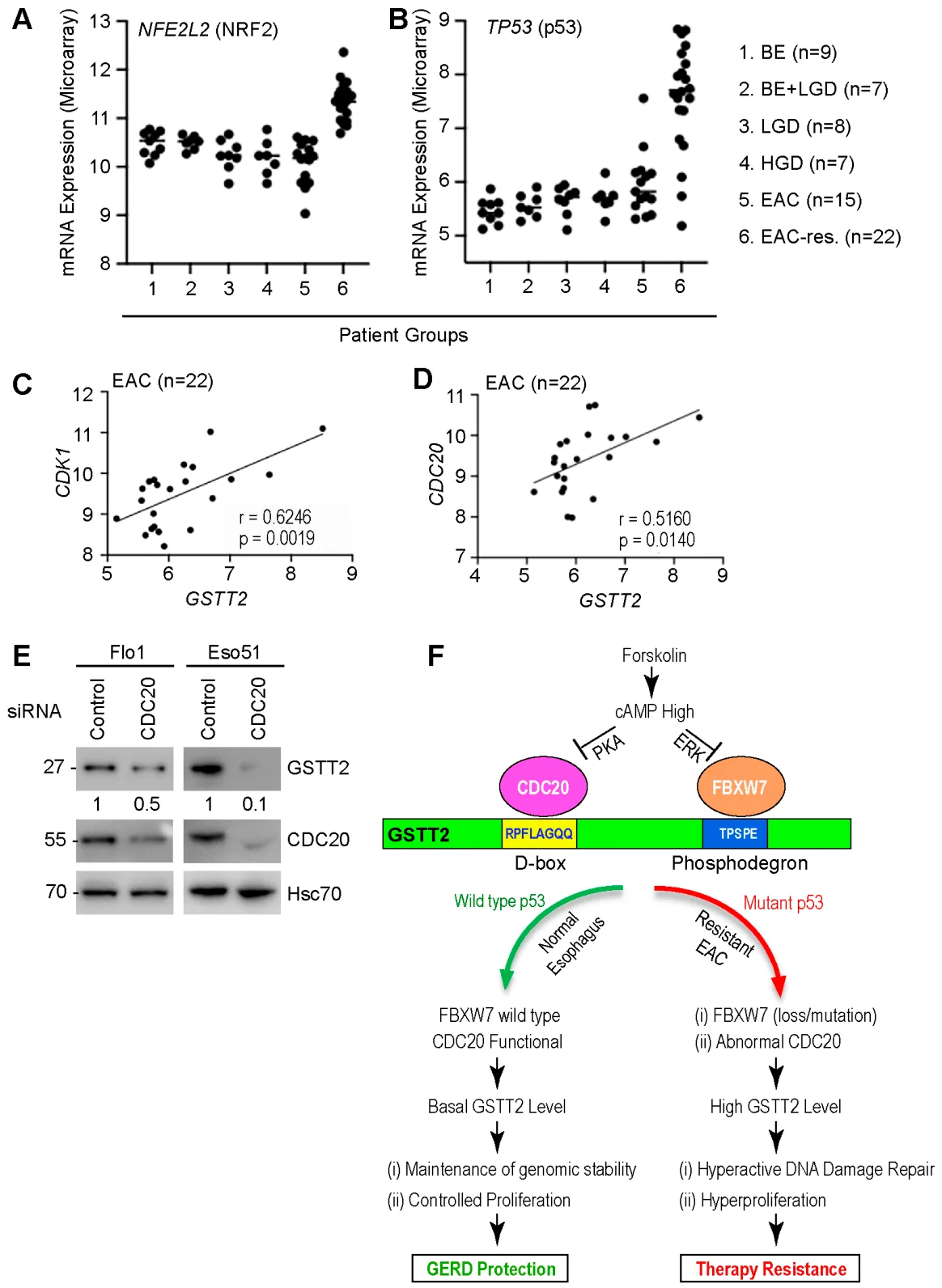
**In treatment-resistant EACs, GSTT2 expression is induced and is correlated with mitotic regulators.** (**A**) Affymetrix-based gene expression of *GSTT2*, *NFE2L2* and *TP53* was analyzed in samples isolated from six different groups (Barrett’s esophagus (BE), low- and high-grade dysplasia (LGD, HGD), EAC and treatment-resistant EAC, as shown. (**B**–**E**) We correlated gene expression between *GSTT2* and *CDK1* and between *GSTT2* and *CDC20* in treatment-resistant EAC patients (*n* = 22) and in an independent cohort of EAC/GEJ tumors (*n* = 122), as indicated. Analyses include Pearson correlation (r value) and statistical significance (*p*-value) for each gene set. (**F**) Two EAC cell lines (Flo1 and Eso51) were transfected with either control or CDC20 siRNAs. Forty-eight hours post-transfection, cell lysates were prepared and subjected to immunoblotting using the indicated antibodies. Schematic model showing, (i) in the normal esophagus, the importance of the FBXW7/CDC20-GSTT2 axis involved in degrading GSTT2 protein levels in wild-type p53-containing normal esophageal cells, maintaining cellular homeostasis by protecting cells from redox-induced DNA damage repair, and, (ii) in contrast, in EAC cells with mutant p53 and additional presence of *FBXW7* loss or mutation, causing aberrant CDC20 expression/activation and stabilizing GSTT2 protein to promote efficient DNA damage repair and hyperproliferative potential, causing therapy resistance.

**GSTT2 expression increases in treatment-resistant EAC and correlates with mitotic markers.** Using microarray-based gene expression data from Barrett’s esophagus (BE), low- and high-grade dysplasia (LGD and HGD), esophageal adenocarcinoma (EAC), and treatment-resistant EAC samples, we observed a gradual increase in *GSTT2* expression in the treatment-resistant EAC group (Supplemental Figure S1A). Previous studies have identified an antioxidant response element (ARE) within the *GSTT2* gene, which is regulated by the transcription factor NRF2 [33]. Our gene expression dataset revealed increased NRF2 expression specifically in treatment-resistant EAC patients (Figure 6A); further correlation studies did not show significance at the transcript level (r = −0.04; *p* = 0.82) (Supplemental Figure S3G). Because the majority of EACs harbor *TP53* mutations and mutant p53 has been reported to exert oncogenic effects by binding to NRF2, forming a heteromeric transcription factor and activating downstream redox-regulatory targets that promote therapy resistance [45], we next examined *TP53* expression. We noticed a gradual increase in *TP53* expression from BE to EAC but a substantial increase in treatment-resistant EAC samples (Figure 6B). Although *GSTT2*, *NRF2*, and *TP53* all showed increased expression trends in treatment-resistant EAC, Pearson correlation analysis did not reveal significant correlations between *GSTT2* and *NRF2* (Supplemental Figure S3G). At the transcript level, *GSTT2* expression in treatment-resistant EAC was most strongly correlated with *CDK1*, a key cyclin-dependent kinase that drives mitotic progression (Figure 6C). This correlation between *GSTT2* and *CDK1/CDC20* flips in treatment-naïve EAC patients, as they are inversely correlated (*n* = 122; Supplemental Figure S2). Interestingly, *CDC20*, another critical regulator of mitosis, was also significantly correlated with *GSTT2* expression (Figure 6D). This pattern differed from our observations in normal esophageal cells, where *CDC20* appeared to function as a regulator of GSTT2 degradation. In EAC cell lines, including Flo-1 and ESO51 cells, we observed loss of GSTT2 protein levels upon *CDC20* knockdown, suggesting potential oncogenic cooperation between the CDC20-GSTT2 axis during progression to cancer (Figure 6E). Together, these findings suggest that GSTT2 may switch from its protective role in normal esophageal tissue to promoting cell survival in therapy-resistant cancer cells. This oncogenic function may involve cooperation with mutant p53 and CDC20, possibly in the context of impaired FBXW7 E3 ligase activity caused by allele loss or missense mutation. Such alterations may promote hyperproliferation, enhance DNA damage response capacity, and ultimately contribute to therapy resistance in EAC (Figure 6F). This observation raised an yet unanswered question: how does CDC20 switch from a degrader to a stabilizer of GSTT2 protein during Barrett’s to EAC progression, and what are the possible modifiers of cancer-associated signaling or post-translational modifications that alter the relationship?

## 4. Discussion

In this study, we define the cellular distribution and regulatory features of GSTT2 in the human esophagus and identify a potential context-dependent role for GSTT2 in epithelial homeostasis, cell-cycle regulation, and EAC therapy resistance. Building on our prior observation that *GSTT2* transcript and protein levels are higher in AA compared with EA individuals, we show here that GSTT2 protein is broadly expressed throughout the human esophageal epithelium, including p63-positive basal/progenitor cells, with higher expression in dysplastic BE regions. Mechanistically, we identify GSTT2 as a cell-cycle-regulated protein whose abundance is influenced by CDC20, CDH1, FBXW7, and forskolin-sensitive signaling. Finally, analysis of patient gene expression datasets indicates that GSTT2 expression increases in treatment-resistant EACs, where it correlates with mitotic regulators including CDK1 and CDC20. Together, these findings support a model in which GSTT2 contributes to normal epithelial stress response programs but may be co-opted during therapy resistance, promoting better DNA damage response and hyperproliferation.

A key finding of this work is that GSTT2 expression is not restricted to a single epithelial compartment. FISH and immunofluorescence analyses demonstrated *GSTT2* mRNA and protein throughout the stratified squamous epithelium, extending from the basal layer to the luminal surface, with additional signal in the lamina propria. Its presence in p63-positive basal cells is particularly notable because this compartment contains progenitor populations responsible for epithelial maintenance and regeneration [46,47]. These data suggest that GSTT2 may support redox homeostasis or xenobiotic defense not only in differentiated epithelial cells exposed to luminal injury but also in long-lived progenitor cells that must preserve genomic integrity over time. GSTT2 expression in the developing esophagus further suggests that its activity is established early during epithelial maturation and may represent a conserved protective feature of esophageal tissue biology.

Our spatial analyses also elucidate the cellular basis of previously observed ancestry-associated differences in GSTT2 expression [10]. The overall localization pattern of GSTT2 was similar between AA and EA tissues, suggesting that these differences reflect variation in expression level rather than the occurrence of a distinct GSTT2-positive cell population. This distinction is important because it supports the idea that GSTT2 is part of a shared epithelial program whose baseline activity varies among individuals or populations. Such variation may arise from genetic, epigenetic, environmental, dietary, inflammatory, and microbiome-related factors, as well as interactions among these factors. Therefore, the observed AA and EA differences should not be interpreted as purely genetic or biologically deterministic, but rather as a foundation for future studies investigating mechanisms that regulate GSTT2 expression across diverse populations.

A central and somewhat unexpected finding of this study is that *GSTT2* mRNA is not progressively induced along the metaplasia-to-dysplasia-to-adenocarcinoma sequence. On a platform-independent percentile axis, *GSTT2* mRNA was highest in the normal squamous esophagus, declined across the BE-to-EAC progression, and was much more highly expressed in therapy-resistant tumors. The direction of the early change was corroborated in an independent esophageal/gastroesophageal cohort, in which GSTT2 was significantly higher in normal squamous epithelium than in adenocarcinomas. This trajectory suggests that *GSTT2* is a gene whose high baseline expression is a property of normal squamous tissue rather than a marker acquired during transformation. Such a pattern is consistent with our spatial data placing GSTT2 in the p63-positive basal/progenitor population: as the epithelium is replaced by intestinalized columnar metaplasia and subsequently by adenocarcinoma, the squamous progenitor programs that sustain high GSTT2 are lost, and average GSTT2 expression falls accordingly. Loss of a squamous antioxidant enzyme during columnar transformation may itself be permissive for disease because it would be expected to lower the epithelium’s safeguarding capacity against the reflux-associated oxidative stress that drives BE pathogenesis [48,49,50]. Notably, all these analyses were performed at the transcript level; because we observed foci of dysplasia within BE tissue that exhibited elevated GSTT2 protein (Figure 1D), further evaluation of GSTT2 at the protein level across the BE-to-EAC progression sequence will be important.

Beyond its localization and levels of expression, our data identify GSTT2 as a cell-cycle-regulated protein. Sequence analysis revealed putative APC-C [41] and FBXW7-recognition motifs [51], and synchronized cell-cycle studies showed dynamic GSTT2 expression, with lower levels during S-to-G2/M and increased abundance during mitotic exit and early G1. Subcellular fractionation demonstrated similar cell-cycle-dependent GSTT2 kinetics in cytosolic and membrane fractions, with little detectable nuclear GSTT2 under the conditions tested. These findings suggest that GSTT2 abundance is coordinated with proliferative state, potentially linking redox regulation, protein turnover, and cell-cycle progression.

The third major observation is that the transcriptional pathways associated with GSTT2 change qualitatively with disease state. In the normal esophagus, genes co-expressed with GSTT2 yielded no pathway surviving multiple testing correction, but the oxidative stress pathway was observed. In the BE-to-EAC progression cohort with resistant tumors excluded, only three terms were significant, none implicating the cell cycle. In therapy-resistant EAC, however, the *GSTT2* co-expression network was overwhelmingly proliferative, dominated by the G2-M checkpoint, E2F targets, and Reactome Cell Cycle, with leading-edge mitotic regulators including *CDK1*, *CDC20*, *CCNB1*, *BUB1*, and *UBE2C*. The count of significantly enriched terms rose from none in normal esophageal tissue to hundreds in resistant EACs, and this signal was contributed almost entirely by the 22 resistant specimens rather than by the other tumors. We interpret this as a resistance-specific functional switch, in which *GSTT2* transitions from its homeostatic role in the quiescent squamous epithelium to becoming coupled to the mitotic program of aggressive, treatment-refractory tumors.

This co-expression switch is mechanistically coherent with the post-translational regulation we describe. The relationship between GSTT2 and APC-C regulation appears complex. GSTT2 was detected in immunocomplexes containing both APC-C co-activators CDC20 and CDH1, yet these factors had opposing effects on GSTT2 abundance. CDC20 overexpression reduced GSTT2 levels, whereas CDC20 loss increased GSTT2, supporting a model in which APC-CDC20 promotes GSTT2 degradation during G2/M. In contrast, CDH1 overexpression increased GSTT2 levels, while CDH1 loss decreased GSTT2, suggesting that CDH1 may stabilize GSTT2 in this context, possibly through suppression of CDC20, modulation of upstream signaling, or regulation of additional GSTT2-targeting factors. FBXW7 also influenced GSTT2 abundance, although its effects were similarly context-dependent. FBXW7 overexpression reduced GSTT2, whereas FBXW7 depletion also destabilized GSTT2, potentially through indirect upregulation of CDC20 [42]. These data suggest that GSTT2 stability is governed by an interconnected regulatory network rather than a single linear E3 ligase pathway.

Forskolin-mediated induction of GSTT2 further supports the idea that GSTT2 turnover is sensitive to signaling pathways that converge on APC-C and FBXW7-associated mechanisms [37,38]. Forskolin increased GSTT2 levels in a dose-dependent manner and rescued CDC20- or FBXW7-associated GSTT2 loss. Functionally, forskolin enhanced clonogenic survival of Het-1A cells, suggesting that GSTT2 induction or related forskolin-responsive pathways may improve epithelial cell fitness. However, forskolin did not significantly enhance repair of hydrogen peroxide-induced DNA damage, and H_2_O_2_-mediated GSTT2 downregulation was not rescued by forskolin. Thus, GSTT2-mediated protection may be context-specific and may involve detoxification, redox buffering, cell-cycle adaptation, or survival signaling rather than direct repair of acute oxidative DNA lesions. In a prior study [52], a cranberry proanthocyanidin (C-PAC) effect was tested on normal primary esophageal cells exposed to a bile acid cocktail, which showed induction of GSTT2 and improved cellular viability.

Analysis of disease progression datasets suggests that GSTT2 may undergo a functional shift during transformation from the normal esophagus to EAC. In normal tissue, GSTT2 expression correlated with oxidative stress response genes, consistent with its canonical role in glutathione-dependent detoxification and redox homeostasis. In contrast, in treatment-resistant EAC, GSTT2-associated pathways were enriched for DNA damage response, repair, and cell-cycle regulation. The positive correlation between GSTT2 and mitotic regulators CDK1 and CDC20 in treatment-resistant EAC is particularly intriguing. In normal cell-cycle models, CDC20 appeared to promote GSTT2 degradation; however, in EAC cells, CDC20 loss reduced GSTT2 levels, suggesting that the CDC20-GSTT2 relationship may be rewired during malignant progression. In cancer cells, CDC20 may cooperate with GSTT2 to support mitotic progression, survival under therapeutic stress, or adaptation to genomic instability. This switch could reflect altered APC-C substrate specificity, changes in post-translational modification, altered proteasomal activity, or broader oncogenic rewiring of cell-cycle checkpoints. These findings support a model in which GSTT2 is protective in normal esophageal epithelium but may acquire pro-tumorigenic or therapy-resistance functions in EAC. As the occurrence of the *TP53* mutation is a frequent and early event [4,5,53] and as wild-type p53 is known to suppress CDC20 expression upon induction of DNA damage [54], future studies may be necessary to understand the role of mutant p53 in hyperactivation of CDC20 and whether such regulation is primarily transcriptional or whether there may be some aspects of post-translational modification of CDC20 influencing its functionality.

Prior studies have suggested that *GSTT2* can be regulated by NRF2, a master transcription factor controlling antioxidant response pathways [33]. In our datasets, however, *GSTT2* did not significantly correlate with *NFE2L2*/*NRF2* across the normal esophagus, BE, dysplasia, chemotherapy-naïve EAC, or therapy-resistant EAC. Although NRF2 expression increased in therapy-resistant EAC, the lack of transcript-level correlation with *GSTT2* suggests that GSTT2 induction in this context may not be driven solely by canonical NRF2-mediated transcription. Instead, our analysis points toward context-specific regulatory programs. In the normal esophagus, *GSTT2* correlated with several transcription factors, most notably *ZBTB32*, whereas in therapy-resistant EAC, GSTT2 correlated strongly with *FOXN2*. These observations suggest that GSTT2 transcriptional control may be rewired during malignant progression and therapy resistance. Because mutant p53 can cooperate with NRF2 and other transcriptional regulators to promote antioxidant and survival programs, it remains possible that GSTT2 is regulated through mutant p53-dependent chromatin remodeling, enhancer activation, or indirect transcriptional networks rather than through NRF2 expression alone [55,56]. To emphasize, neither ZBTB32 nor FOXN2 has been linked to *GSTT2* expression. Importantly, ZBTB32, a zinc finger and BTB domain-containing transcription factor, is a regulator of immune response and metabolic adaptation [57,58]. In contrast, FOXN2 is a known tumor suppressor that is downregulated in breast cancer [59]. Interestingly, prior work from our group reported that chromosome 2p (where *FOXN2* is located) shows frequent chromosomal gains in EAC [60], suggesting a possible link during the development of therapy resistance. Future studies may be necessary to better understand how this context is dependent on GSTT2 biology.

This study has several limitations. The number of human tissue samples analyzed by FISH and immunofluorescence was limited, and larger cohorts will be needed to quantify variation by ancestry, age, sex, disease state, and exposure history. The AA and EA comparisons demonstrate reproducible differences in GSTT2 mRNA and protein abundance but do not establish causality or identify the underlying genetic, epigenetic, environmental, or socioeconomic drivers. Pertaining to induction of *GSTT2* mRNA, there are multiple possibilities, including enhanced transcription, increased mRNA stability, or both. Future studies may be essential to better understand such mechanisms. Mechanistic studies were performed largely in HeLa and Het-1A cells, which are useful experimental models but do not fully represent normal esophageal epithelium or EAC. Validation in patient-derived esophageal organoids, Barrett’s models, and EAC cell lines will be important. In addition, although sequence prediction, co-immunoprecipitation, gain- and loss-of-function experiments, and pharmacologic rescue support APC-C and FBXW7-associated regulation, direct ubiquitination of GSTT2 and degron dependence remain to be demonstrated. Finally, the association between GSTT2 and treatment resistance is correlative, and functional studies are needed to determine whether GSTT2 directly alters chemotherapy, radiation, or targeted therapy response.

In summary, our findings identify GSTT2 as a broadly expressed esophageal protein present in epithelial, basal/progenitor, and non-epithelial compartments. Mechanistically, GSTT2 abundance is dynamically regulated across the cell cycle and influenced by APC-CDC20, -CDH1, and FBXW7-associated pathways. During EAC progression, GSTT2 expression increases and becomes associated with therapy resistance and mitotic regulators. These results support a model (Figure 6F) in which GSTT2 contributes to normal esophageal stress protection but may become integrated into cancer-associated cell-cycle and survival programs during therapy resistance.

## 5. Conclusions

In summary, our study redefines GSTT2 as an ancestry-associated, cell-cycle-regulated protein with epithelial and stromal expression whose function varies across tissue contexts and stages of esophageal cancer progression. GSTT2 is broadly expressed throughout the normal squamous epithelium, including p63-positive basal and progenitor cells, where it is associated with redox and oxidative stress response programs and is most abundant in healthy tissue. Its expression declines during the progression from Barrett’s esophagus to adenocarcinoma but is re-induced in treatment-resistant esophageal adenocarcinoma. In this setting, GSTT2 co-expression shifts from homeostatic antioxidant programs to proliferative and mitotic pathways involving CDK1 and CDC20.

Our biochemical and cell-cycle analyses further show that GSTT2 abundance is regulated by an interconnected network involving APC-CDC20, -CDH1, and FBXW7 and can be pharmacologically stabilized by forskolin. These findings position GSTT2 as a cell-cycle-coupled effector rather than merely a static antioxidant enzyme. Importantly, the ancestry-associated differences observed in this study reflect variation in expression level rather than spatial distribution. Thus, GSTT2 appears to be part of a shared epithelial program whose baseline activity is shaped by genetic, epigenetic, and environmental factors and should not be interpreted as a deterministic ancestry-related trait.

Together, these findings support a model in which a normally protective squamous enzyme is co-opted by the proliferative machinery of aggressive, treatment-refractory tumors. This work provides a foundation for future studies to determine whether GSTT2 causally influences treatment response and to assess its potential as a biomarker and therapeutic target in treatment-resistant esophageal adenocarcinoma.

## Data Availability

All data presented in this manuscript are predominantly included in the main figures, Supplementary Figures S1–S5 and Supplementary Tables S1–S3.

## Supplementary Materials

The following supporting information can be downloaded at https://www.mdpi.com/article/10.3390/cancers18193220/s1, Figure S1: *GSTT2* mRNA across esophageal progression, malignant transformation, and ancestry. (A) *GSTT2* expression, normalized as within-sample percentile rank, across normal esophagus (AA/EA cohort, *n* = 24, Ferrer-Torres et al Gastroenterology 2019) and the GSE37203 progression series: BE (*n* = 9), BE+LGD (*n* = 7), LGD (*n* = 8), HGD (*n* = 7), chemotherapy-naïve EAC (*n* = 15), and therapy-resistant EAC (*n* = 22). *GSTT2* rank is high in normal squamous esophagus, low across progression, and rises specifically in resistant EAC (Kruskal–Wallis *p* = 1.1 × 10^−5^; resistant versus chemotherapy-naïve EAC, Mann–Whitney *p* = 1.2 × 10^−4^). (B) *GSTT2* expression (log_2_) in an independent esophageal/gastroesophageal cohort (Ferrer-Torres, Oncotarget 2016) [31] comprising normal squamous esophagus (*n* = 8), normal gastric mucosa (*n* = 5), and gastroesophageal adenocarcinoma (*n* = 35); *GSTT2* is highest in normal squamous tissue and significantly reduced in adenocarcinomas (Mann–Whitney *p* = 0.013). (C) *GSTT2* in normal esophagus, AA versus EA (log_2_FC = 2.9, Mann–Whitney *p* = 1.7 × 10^−3^), from Ferrer-Torres et al. (2019) [10] at the mRNA level. Boxes show median and interquartile range; points are individual samples; Figure S2: We have performed correlation of gene expression between *GSTT2* and *CDK1*, between *GSTT2* and *CDC20* in treatment naive GEJ (*n* = 70) and EAC (*n* = 52) patients as indicated. Analyses include Pearson correlation (r value) and statistical significance (*p*-value) for each gene set; Figure S3: We have performed correlation of gene expression between GSTT2 and NFE2L2 in different cohorts (normal squamous (NS), Barrett’s (BE), low grade dysplasia (LGD), high grade dysplasia (HGD), in two different cohorts of treatment naïve and also treatment resistant EAC patients as indicated. Analyses include Pearson correlation (r value) and statistical significance (*p*-value) for each gene set; Figure S4: GSTT2–transcription-factor correlations in normal esophagus (Beer Gene ST, *n* = 40), and therapy-resistant EAC (GSE37203, *n* = 22) (A) Top 20 significant transcription factors ranked by correlation coefficient (red, positive; blue, negative). (B) scatter dot plot of the strongest hit, *ZBTB32*, against GSTT2, with samples colored by ancestry/tissue group. (C) Top 20 significant transcription factors ranked by correlation coefficient (red, positive; blue, negative). (D) Scatter dot plot of the FDR-significant hit, *FOXN2*, against GSTT2 (r = +0.82, FDR = 0.003); Figure S5: Expression correlation between GSTT2 and FBXW7. (A) FBXW7 mutation frequency in different human cancers (1: stomach adeno—TCGA—Nature 2014; 2: stomach adeno—TCGA pancancer atlas; 3: esophageal carcinoma—TCGA Firehose legacy; 4: esophageal carcinoma—TCGA Nature 2017; 5: esophageal adeno—TCGA pancancer atlas; 6: stomach adeno—TCGA GDC 2025; 7: gastric cancer—OncoSG 2018; 8: stomach adeno—Pfizer & UHK Nat. Genetics 2014; 9: esophageal—MSK 2023; 10: stomach adeno—UHK Nat. Genetics 2011; 11: esophagogastric cancer—MSK Clinc. Cancer Res. 2022; 12: metastatic esophagogastric—MSK Cancer Disco. 2017; 13: esp[hageal/stomack—MSK 2020; 14: esophageal squamous—UCLA Nat. Genetics 2014; 15: esophageal squamous—ICGC Nature 2014; 16: gastric adeno—TMUCIH PNAS 2015; 17: esophageal adeno—DFCI Nat.Genetics 2013; 18: esophagogastric—MSK Clinc. Cancer Res. 2023; 19: esophageal cancer—TRAP project—Lancet 2020. (B) Gene expression between *GSTT2* and *FBXW7 in treatment resistant EAC* patients. Analyses include Pearson correlation (r value) and statistical significance (*p*-value). (C,D) Immunofluorescence staining of GSST2; Table S1: Pathways associated with GSTT2 in normal esophagus (*n* = 40; 44 protein-coding co-expressed genes); Table S2: Pathways associated with GSTT2 in Barrett’s–EAC progression only, excluding therapy-resistant tumors (*n* = 46; 113 co-expressed genes). Top 15 terms ranked by *p*-value; Table S3: Pathways associated with GSTT2 in therapy-resistant EAC only (*n* = 22; 691 co-expressed genes). Top 20 FDR-significant terms.

## Abbreviations

The following abbreviations are used in this manuscript:

AA	African American
EA	European American
BE	Barrett’s esophagus
LGD	Low-grade dysplasia
HGD	High-grade dysplasia
EAC	Esophageal adenocarcinoma
GSTT2	Glutathione S-transferase theta 2
S.D.	Standard deviation
