# Supplementary material for: GSTT2 Switches from a Homeostatic Antioxidant to a Cell-Cycle-Coupled Factor in Therapy-Resistant Esophageal Adenocarcinoma
Source: Cancers (Basel). 2026 Oct 6;18(19):3220. doi: 10.3390/cancers18193220 (PMC13664410; doi:10.3390/cancers18193220)
Supplement: Supplementary file 1 [file cancers-18-03220-s001.zip › cancers-4535164-supplementary.pdf]

Supplemental Table S1. Pathways associated with GSTT2 in normal esophagus (n = 40; 44 protein-coding co-expressed genes).

Library	Pathway	Overlap	P-value	FDR	Representative genes
**GO-BP**	Adenylate Cyclase-Activating Adrenergic Receptor Signaling Pathway	2/15	4.9e-04	0.09	*ADRA1B;GPR101*
**GO-BP**	Adrenergic Receptor Signaling Pathway	2/18	7.1e-04	0.09	*ADRA1B;GPR101*
**GO-BP**	Sensory Perception Of Mechanical Stimulus	3/89	9.9e-04	0.09	*MYH7B;ESPNL;EML2*
**GO-BP**	Sensory Perception Of Sound	3/92	1.1e-03	0.09	*MYH7B;ESPNL;EML2*
**GO-BP**	Regulation Of Microtubule Polymerization	2/40	3.5e-03	0.13	*CAV3;EML2*
**Reactome**	Activation Of Gene Expression By SREBF (SREBP)	2/42	3.9e-03	0.28	*NCOA6;PKMYT1*
**Reactome**	Smooth Muscle Contraction	2/43	4.0e-03	0.28	*MYL7;CAV3*
**Hallmark**	Apical Surface	2/44	4.2e-03	0.06	*NCOA6;IL2RB*
**GO-BP**	Natural Killer Cell Activation	2/45	4.4e-03	0.13	*NCR3;IL2RB*
**GO-BP**	Receptor Internalization	2/51	5.6e-03	0.13	*CAV3;ITGB2*
**Reactome**	Regulation Of Cholesterol Biosynthesis By SREBP (SREBF)	2/55	6.5e-03	0.28	*NCOA6;PKMYT1*
**GO-BP**	Spliceosomal Complex Assembly	2/62	8.2e-03	0.13	*NR5A1;DDX39B*
Redox / oxidative stress and DNA-damage / repair terms					
**GO-BP**	Positive Regulation Of Superoxide Anion Generation	1/13	2.8e-02	0.13	*ITGB2*
**GO-BP**	Regulation Of Superoxide Anion Generation	1/14	3.0e-02	0.13	*ITGB2*
**GO-BP**	Positive Regulation Of Reactive Oxygen Species Metabolic Process	1/48	1.0e-01	0.18	*ITGB2*
**Reactome**	Biological Oxidations	1/218	3.8e-01	0.52	*UGT3A1*
**GO-BP**	Double-Strand Break Repair Via Synthesis-Dependent Strand Annealing	1/5	1.1e-02	0.13	*XRCC3*
**GO-BP**	Regulation Of DNA Damage Checkpoint	1/18	3.9e-02	0.14	*DDX39B*
**KEGG**	Homologous recombination	1/41	8.6e-02	0.41	*XRCC3*
**Reactome**	HDR Thru Homologous Recombination (HRR)	1/67	1.4e-01	0.35	*XRCC3*
**GO-BP**	DNA Damage Response	2/384	2.1e-01	0.27	*NCOA6;XRCC3*

Pathways enriched among genes co-expressed with GSTT2 (Pearson $|r| \geq 0.5$) in each cohort, by over-representation analysis (Enrichr) against GO, KEGG, Reactome, WikiPathways, and MSigDB Hallmark libraries. *P*-values are Fisher exact; FDR is Benjamini–Hochberg across all tested terms within a cohort.

Supplemental Table S2. Pathways associated with GSTT2 in Barrett's–EAC progression only, excluding therapy-resistant tumors (n = 46; 113 co-expressed genes). Top 15 terms ranked by P-value.

Library	Pathway	Overlap	P-value	FDR	Representative genes
**Reactome**	TFAP2 (AP-2) Family Regulates Transcription Of Growth Factors And Their Receptors	3/13	4.8e-05	0.018	*YY1;TFAP2C;ERBB2*
**Hallmark**	UV Response Dn	6/144	1.7e-04	0.007	*MMP16;DLC1;ERBB2;ICA1;TFPI;MAPK14*
**GO-CC**	Basolateral Plasma Membrane	6/171	4.2e-04	0.044	*NDRG4;B4GALT1;ERBB2;SLC4A10;SLC29A2;AURKA*
**KEGG**	Non-small cell lung cancer	4/72	7.4e-04	0.077	*RET;ERBB2;STK4;DDB2*
**WikiPathways**	Non Small Cell Lung Cancer	4/72	7.4e-04	0.192	*CRABP1;ERBB2;STK4;DDB2*
**GO-BP**	Cellular Response To UV	4/79	1.0e-03	0.270	*YY1;MAPK14;SPAM1;DDB2*
**Reactome**	Transcriptional Regulation By AP-2 (TFAP2) Family Of Transcription Factors	3/36	1.1e-03	0.148	*YY1;TFAP2C;ERBB2*
**GO-BP**	Calcium Ion Transport Into Cytosol	2/9	1.1e-03	0.270	*CACNA2D1;SLC8A1*
**Reactome**	Interaction With Cumulus Cells And Zona Pellucida	2/10	1.4e-03	0.148	*B4GALT1;SPAM1*
**GO-BP**	Fatty Acid Beta-Oxidation Using acyl-CoA Dehydrogenase	2/10	1.4e-03	0.270	*ACADL;ETFA*
**Reactome**	CDC42 GTPase Cycle	5/149	1.6e-03	0.148	*DOCK9;DLC1;ARHGDIG;ARHGAP1;BAIAP2*
**GO-BP**	Heart Contraction	3/41	1.6e-03	0.270	*SGCD;TNNC1;SLC8A1*
**GO-BP**	Cellular Response To UV-B	2/11	1.7e-03	0.270	*MAPK14;SPAM1*
**KEGG**	Hypertrophic cardiomyopathy	4/90	1.7e-03	0.077	*SGCD;CACNA2D1;TNNC1;SLC8A1*
**WikiPathways**	Vasopressin Regulated Water Reabsorption	3/44	2.0e-03	0.255	*DCTN1;ARHGDIG;DYNC1I1*

Supplemental Table S3. Pathways associated with GSTT2 in therapy-resistant EAC only (n = 22; 691 co-expressed genes). Top 20 FDR-significant terms.

Library	Pathway	Overlap	P-value	FDR	Representative genes
**GO-CC**	Nucleus	297/4487	1.5e-34	4.1e-32	*POP7;RTCA;CCNF;HNRNPR;ENO1;IFI35 (+291)*
**Hallmark**	G2-M Checkpoint	54/200	4.2e-33	2.0e-31	*H2AX;CCNF;SMC4;HOXC10;KIF15;LMNB1 (+48)*
**Hallmark**	E2F Targets	52/200	5.0e-31	1.2e-29	*H2AX;POP7;SMC3;SMC4;CKS1B;LMNB1 (+46)*
**GO-CC**	Intracellular Membrane-Bounded Organelle	319/5175	1.0e-31	1.4e-29	*POP7;RTCA;CCNF;HNRNPR;ENO1;IFI35 (+313)*
**Reactome**	Cell Cycle	93/654	5.0e-32	6.1e-29	*H2AX;ERCC6L;NCAPG2;GMNN;HJURP;MCM10 (+87)*
**Reactome**	Cell Cycle, Mitotic	79/523	5.0e-29	3.1e-26	*H2AX;ERCC6L;NCAPG2;GMNN;MCM10;SMC3 (+73)*
**Reactome**	M Phase	58/380	1.0e-21	4.0e-19	*H2AX;ERCC6L;NCAPG2;SMC3;SMC4;LMNB1 (+52)*
**Reactome**	Cell Cycle Checkpoints	45/271	1.5e-18	4.5e-16	*H2AX;ERCC6L;MCM10;CENPA;ANAPC10;SKA1 (+39)*
**Reactome**	Mitotic Anaphase	41/232	5.1e-18	1.2e-15	*NUP205;ERCC6L;SMC3;PDS5A;CENPA;ANAPC10 (+35)*
**Reactome**	Mitotic Metaphase And Anaphase	41/233	6.0e-18	1.2e-15	*NUP205;ERCC6L;SMC3;PDS5A;CENPA;ANAPC10 (+35)*
**Reactome**	Mitotic Prometaphase	35/186	1.9e-16	3.3e-14	*ERCC6L;NCAPG;SMC3;PDS5A;SMC4;CENPA (+29)*
**Reactome**	Resolution Of Sister Chromatid Cohesion	25/106	1.7e-14	2.6e-12	*ERCC6L;SMC3;PDS5A;CENPA;SKA1;CDC20 (+19)*
**Reactome**	Separation Of Sister Chromatids	30/170	1.6e-13	2.2e-11	*ERCC6L;SMC3;PDS5A;CENPA;ANAPC10;SKA1 (+24)*
**Reactome**	Mitotic Spindle Checkpoint	23/110	2.9e-12	3.5e-10	*ERCC6L;UBE2C;UBE2E1;CENPA;ANAPC10;SKA1 (+17)*
**GO-MF**	RNA Binding	101/1411	1.6e-12	8.3e-10	*SRP19;POP7;RTCA;FCF1;HNRNPR;ENO1 (+95)*
**GO-CC**	Intracellular Non-Membrane-Bounded Organelle	88/1195	1.2e-11	1.1e-09	*FHOD3;SRP19;RBM28;POP7;DDX47;HJURP (+82)*
**Reactome**	Signal Transduction	146/2465	2.3e-11	2.6e-09	*ERCC6L;IRS1;NAB2;PMCH;RBPJ;SMC3 (+140)*
**Reactome**	Unattached Kinetochores Signal Amplification Via A MAD2 Inhibitory Signal	20/93	4.5e-11	4.5e-09	*ERCC6L;CENPA;SKA1;CDC20;PPP2CA;PPP2CB (+14)*
**GO-CC**	Nuclear Lumen	64/780	1.3e-10	8.7e-09	*SRP19;RBM28;POP7;DDX47;HJURP;FCF1 (+58)*
**Reactome**	EML4 And NUDC In Mitotic Spindle Formation	20/97	1.0e-10	8.9e-09	*ERCC6L;CENPA;SKA1;CDC20;PPP2CA;PPP2CB (+14)*

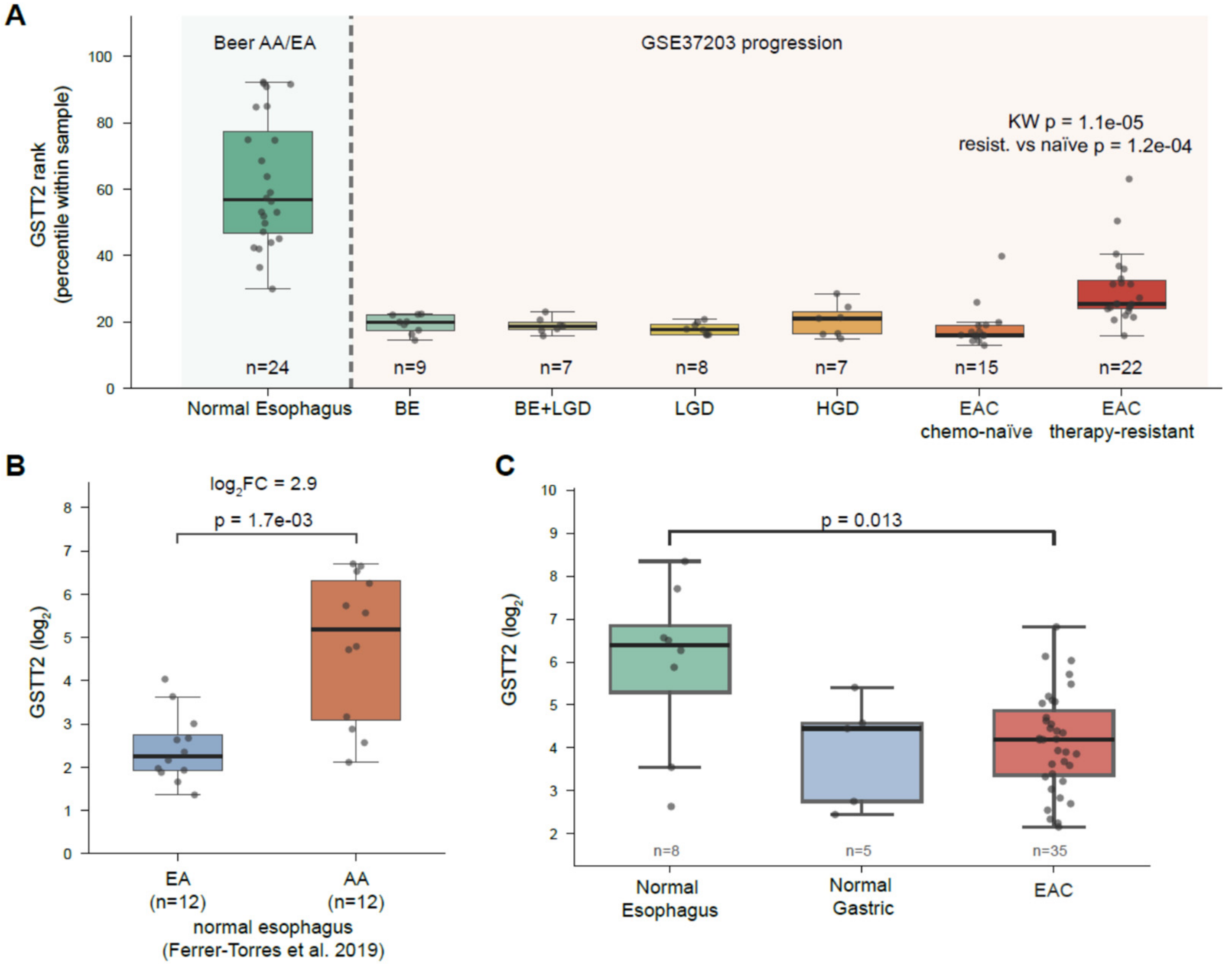

Supplemental Figure S1. *GSTT2* mRNA across esophageal progression, malignant transformation, and ancestry.

(A) *GSTT2* expression, normalized as within-sample percentile rank, across normal esophagus (AA/EA cohort, n = 24, Ferrer-Torres et al Gastroenterology 2019) and the GSE37203 progression series: BE (n = 9), BE+LGD (n = 7), LGD (n = 8), HGD (n = 7), chemotherapy-naïve EAC (n = 15), and therapy-resistant EAC (n = 22). *GSTT2* rank is high in normal squamous esophagus, low across progression, and rises specifically in resistant EAC (Kruskal–Wallis $p = 1.1 \times 10^{-5}$; resistant versus chemotherapy-naïve EAC, Mann–Whitney $p = 1.2 \times 10^{-4}$).

(B) *GSTT2* expression ($\log_2$) in an independent esophageal/gastroesophageal cohort (Ferrer-Torres, Oncotarget 2016) comprising normal squamous esophagus (n = 8), normal gastric mucosa (n = 5), and gastroesophageal adenocarcinoma (n = 35); *GSTT2* is highest in normal squamous tissue and significantly reduced in adenocarcinomas (Mann–Whitney $p = 0.013$).

(C) *GSTT2* in normal esophagus, AA versus EA ($\log_2$FC = 2.9, Mann–Whitney $p = 1.7 \times 10^{-3}$), from Ferrer-Torres et al. (2019) at the mRNA level. Boxes show median and interquartile range; points are individual samples.

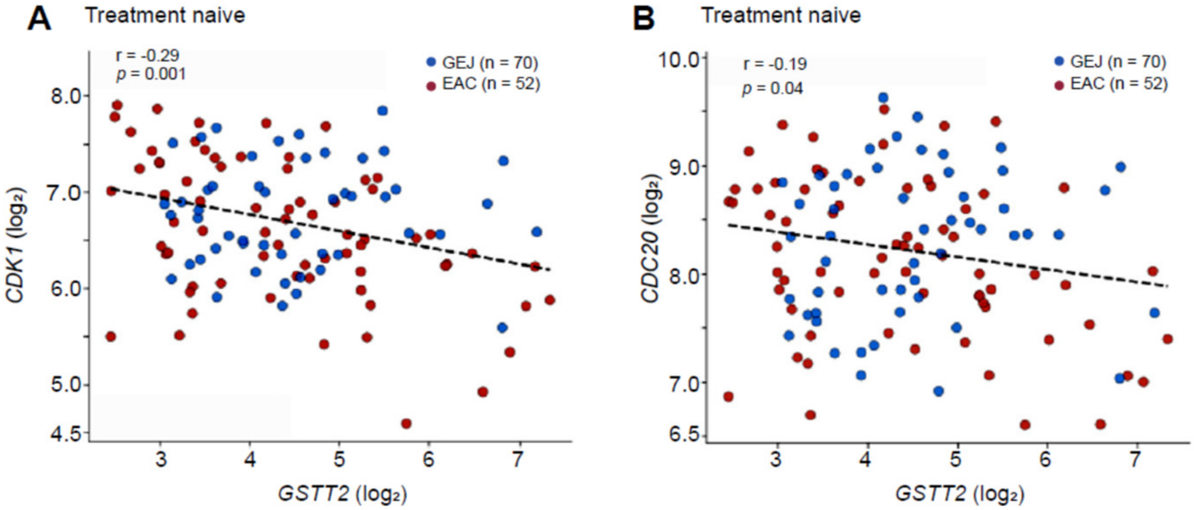

Supplemental Figure S2. We have performed correlation of gene expression between *GSTT2* and *CDK1*, between *GSTT2* and *CDC20* in treatment naive GEJ (n=70) and EAC (n=52) patients as indicated. Analyses include Pearson correlation (r value) and statistical significance (*p*-value) for each gene set.

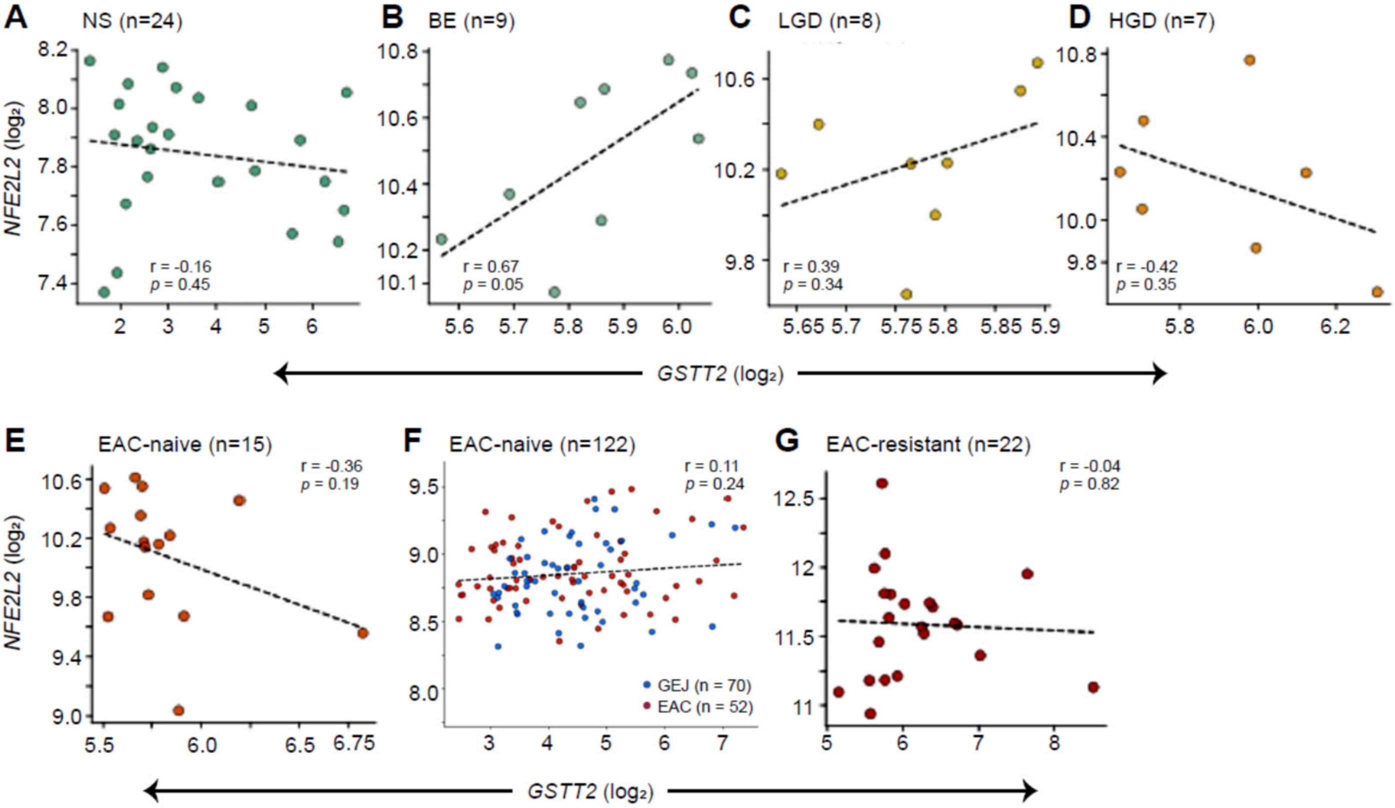

Supplemental Figure S3. We have performed correlation of gene expression between GSTT2 and NFE2L2 in different cohorts (normal squamous (NS), Barrett's (BE), low grade dysplasia (LGD), high grade dysplasia (HGD), in two different cohorts of treatment naïve and also treatment resistant EAC patients as indicated. Analyses include Pearson correlation (r value) and statistical significance (*p*-value) for each gene set.

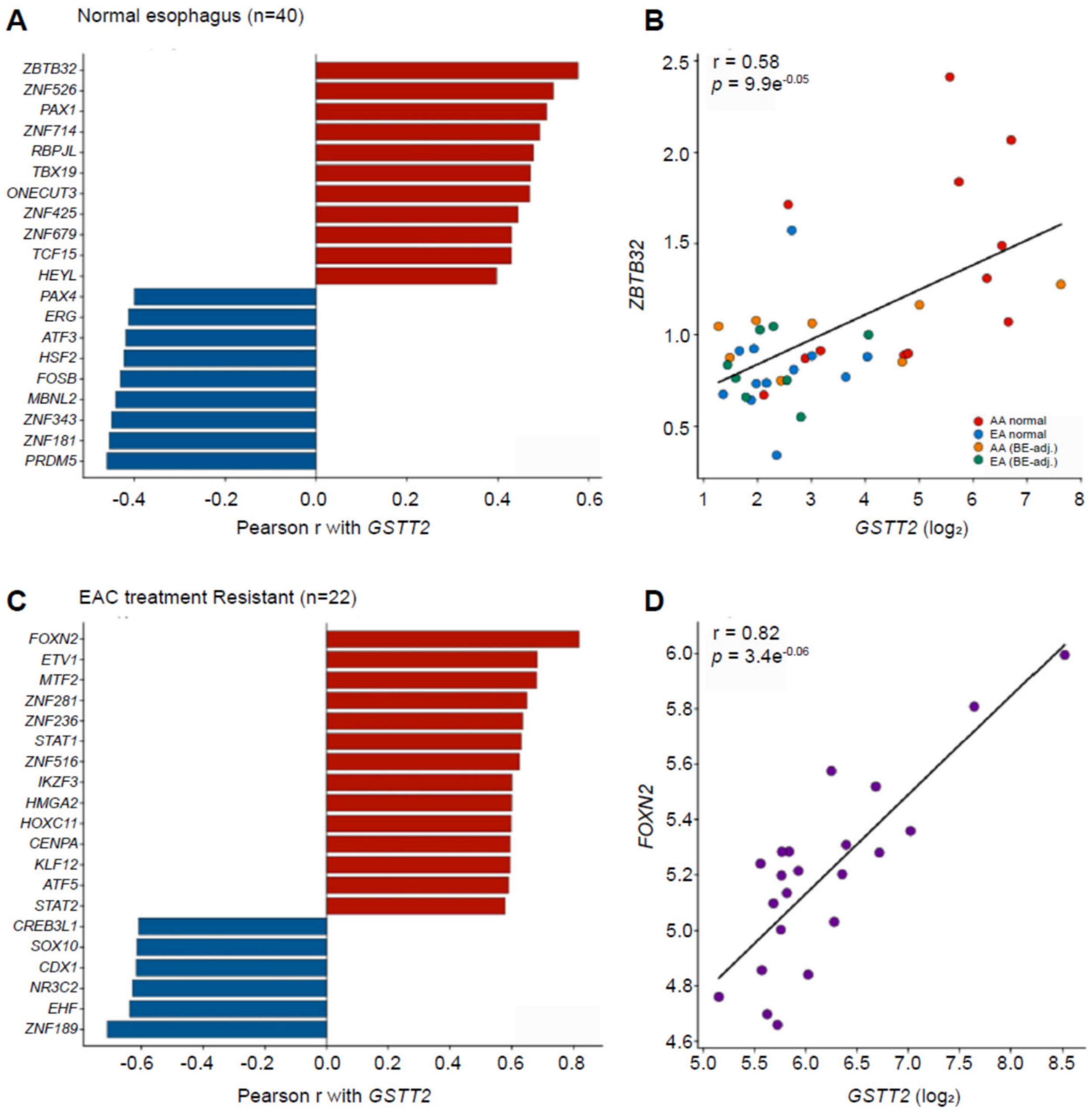

Supplemental Figure S4. GSTT2–transcription-factor correlations in normal esophagus (Beer Gene ST, n = 40), and therapy-resistant EAC (GSE37203, n = 22) (A) Top 20 significant transcription factors ranked by correlation coefficient (red, positive; blue, negative). (B) scatter dot plot of the strongest hit, *ZBTB32*, against GSTT2, with samples colored by ancestry/tissue group. (C) Top 20 significant transcription factors ranked by correlation coefficient (red, positive; blue, negative). (D) Scatter dot plot of the FDR-significant hit, *FOXN2*, against GSTT2 (r = +0.82, FDR = 0.003).

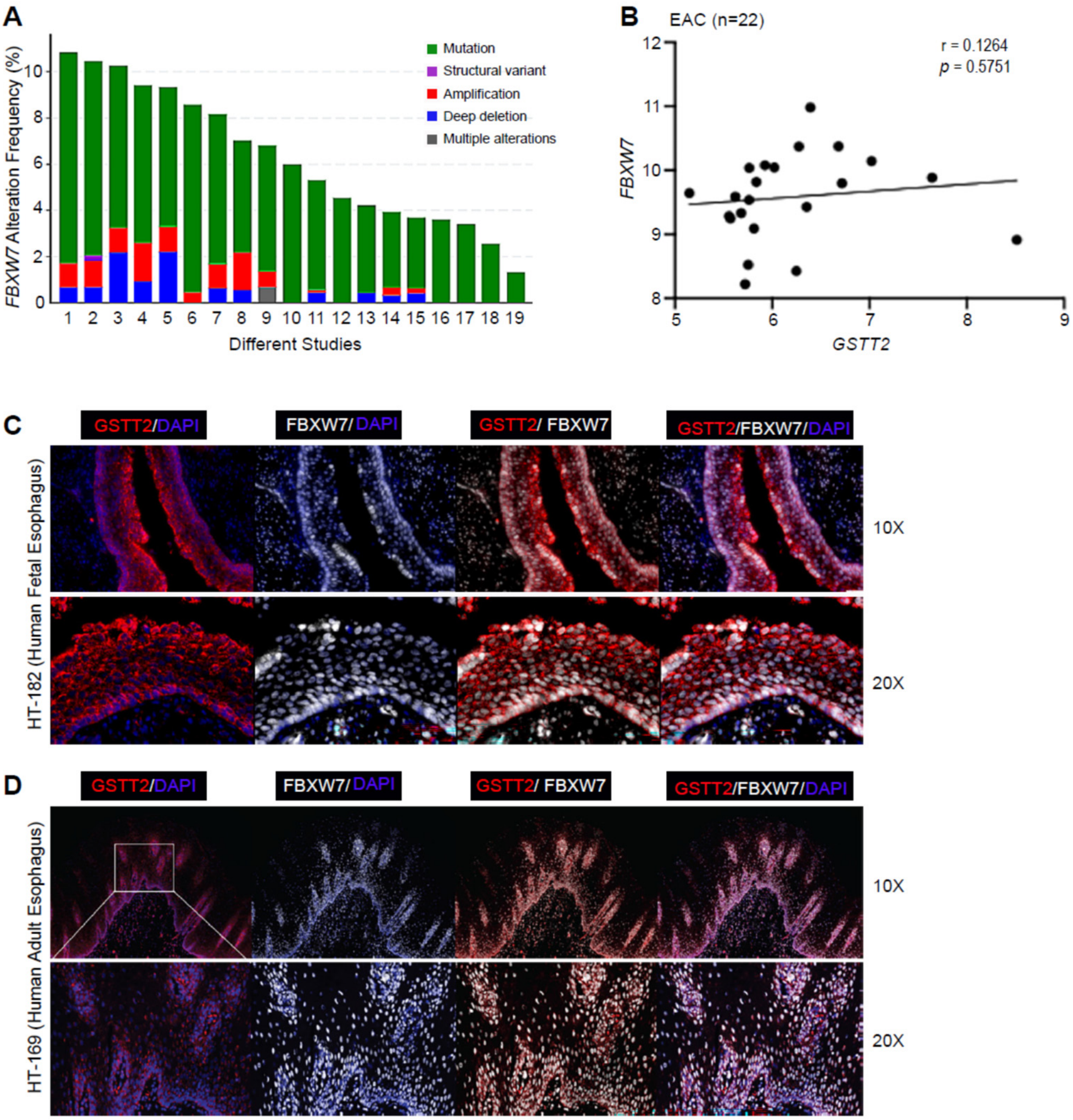

Supplemental Figure S5. Expression correlation between GSTT2 and FBXW7. (A) FBXW7 mutation frequency in different human cancers (1: stomach adeno – TCGA – Nature 2014; 2: stomach adeno – TCGA pancancer atlas; 3: esophageal carcinoma – TCGA Firehose legacy; 4: esophageal carcinoma – TCGA Nature 2017; 5: esophageal adeno – TCGA pancancer atlas; 6: stomach adeno – TCGA GDC 2025; 7: gastric cancer – OncoSG 2018; 8: stomach adeno – Pfizer & UHK Nat. Genetics 2014; 9: esophageal – MSK 2023; 10: stomach adeno – UHK Nat. Genetics 2011; 11: esophagogastric cancer – MSK Clinc. Cancer Res. 2022; 12: metastatic esophagogastric – MSK Cancer Disco. 2017; 13: esp[hageal/stomack – MSK 2020; 14: esophageal squamous – UCLA Nat. Genetics 2014; 15: esophageal squamous – ICGC Nature 2014; 16: gastric adeno – TMUCIH PNAS 2015; 17: esophageal adeno – DFCI Nat.Genetics 2013; 18: esophagogastric – MSK Clinc. Cancer Res. 2023; 19: esophageal cancer – TRAP project – Lancet 2020. **(B)** Gene expression between *GSTT2* and *FBXW7 in treatment resistant EAC* patients. Analyses include Pearson correlation (r value) and statistical significance (p-value). (C & D) Immunofluorescence staining of GSST2 (red) and FBXW7 (white) of human fetal (HT-182) and human adult (HT-169) esophagus.
